# Supplementary material for: A nonparametric statistical method for deconvolving densities in the analysis of proteomic data
Source: BMC Bioinformatics. 2026 Mar 5;27:83. doi: 10.1186/s12859-026-06408-0 (PMC13063680; doi:10.1186/s12859-026-06408-0)
Supplement: Supplementary file 1 — Supplementary file S1 [file 12859_2026_6408_MOESM1_ESM.pdf]

Supplementary file S1

A nonparametric statistical method for deconvolving densities in the analysis of proteomic data

Akin Anarat[1,2], Jean Krutmann[2], and Holger Schwender[1]

[1] *Mathematical Institute, Heinrich Heine University, Düsseldorf, Germany*
[2] *IUF – Leibniz Research Institute for Environmental Medicine, Düsseldorf, Germany*
[*] *Corresponding author: akin.anarat@hhu.de*

This supplementary file provides additional material supporting the main manuscript.

In Section S1, we provide a detailed discussion of the numerical challenges arising in the nonparametric deconvolution of densities. We explain how the smoothness of the convolving distribution and the variance ratio between the involved distributions affect the stability of the deconvolution, and outline existing strategies to mitigate these issues. Moreover, the proof of equation (9) of the main manuscript is provided in this section.

Section S2 presents a detailed description of the established deconvolution methods mentioned in the main manuscript. This includes their theoretical foundations, key assumptions, and practical implementation aspects.

Section S3 provides additional technical details of the NPFD procedure. In Section S3.1, we describe the procedure for the estimation of the density functions of the observed data in the NPFD procedure. Section S3.2 then outlines how the corresponding Fourier transforms are obtained in NPFD based on these density estimates. Together, these sections provide full details of both steps, including the histogram-based Poisson regression approach and the subsequent numerical integration procedure. In Section S3.3 the technical role of the threshold value ε in the choice of N and practical diagnostics for assessing the adequacy of ε are discussed. Section S3.4 outlines the algorithm used to determine the power N. In Section S3.5, we derive the asymptotical behavior of NPFD in N based on empirical Fourier transforms.

In Section S4, the results of the simulation scenarios not included in the main text of the simulation study are presented. Specifically, in Section S4.1 an illustrative example of the NPFD behavior in the Fourier domain across different power values N is visualized and discussed. Section S4.2 provides the figures that display the additional results of the scenarios considered in the comparison of NPFD with FDD proposed by Diggle and Hall [1]. In Section S4.3, the comparison with the approach proposed by Wang and Wang [2] is provided. Section S4.4 includes simulated examples from the comparison with the deconvolution method of Neumann [3].

Finally, in Section S5, Figure S11 illustrates the results of the application of FDD to the proteomic data from the GerontoSys study [4].

S1 Numerical Challenges in Density Deconvolution

The numerical instabilities described in Section "General idea of deconvolving densities" of the main article arise from division by small estimated values of $\phi_X(t)$ in the Fourier domain. A particularly problematic situation arises when the Fourier transform of the error distribution decays rapidly and approaches zero in certain regions, leading to severe amplification of estimation errors. The rate of this decay depends on the smoothness of the error distribution and plays a central role in the difficulty of the deconvolution problem. Understanding these properties is essential for developing stable and accurate estimation methods.

It is a well-known property of the Fourier transform of a probability density function that it tends to decay towards zero as the frequency increases [5]. However, when $|\phi_X(t)|$ becomes very small, any small perturbation or noise in $\phi_X(t)$ or $\phi_Z(t)$ can lead to large fluctuations in their ratio, and thus, in $\phi_Y(t)$. This results in a fluctuating behavior at the tails, making the inverse Fourier transform (see equation (4) of the main manuscript) highly sensitive and unstable to small numerical inaccuracies.

When the estimate of the Fourier transform shows substantial fluctuations in the tails, there are two potential approaches. One option is to truncate these tails by choosing an appropriate smoothing function with corresponding integration limits. This process is often discussed in terms of utilizing a kernel function and selecting an appropriate bandwidth [2, 6], which, in this context, determines the shape and spread of the smoothing kernel and thus affects the effective range of integration in the estimated version of equation (4) of the main manuscript. The goal is to suppress the influence of fluctuations beyond a certain cutoff point in order to stabilize the estimation $\widehat{f}_Y$ of the target density f_Y, which can result in a (very) biased, yet smooth estimate. Alternatively, a part of the oscillating tails can be included in the computation, which can lead to a correspondingly erratic estimation of the target density. However, this approach is typically avoided, as in numerically challenging cases, this can not only cause the estimated target density to deviate considerably from the true density f_Y, but also result in an outcome that is far from resembling a proper density function. To enable an appropriate choice for the bandwidth of the kernel in the Fourier inversion step that minimizes information loss and prevents an overly erratic behavior of $\widehat{f}_Y$, it is, therefore, crucial to achieve minimal oscillatory behavior in the estimated Fourier transform $\widehat{\phi}_Y$. These numerical difficulties primarily stem from two main challenges, which are discussed in the following and can be addressed to facilitate the deconvolution process.

S1.1 Impact of smooth convolving distributions

Smooth density functions are characterized by their rapidly decreasing Fourier transforms. In the context of deconvolution problems, the terms smooth and super smooth are used to describe different classes of density functions with varying smoothness properties [7]. These terms refer to the regularity of the Fourier transforms of the density functions. A density function f is considered smooth, if its Fourier transform $\phi(t)$ decays at a polynomial rate, i.e.

$$d_0|t|^{-\beta} \ \leq \ |\phi(t)| \ \leq \ d_1|t|^{-\beta} \qquad \text{as} \ \ t \to \infty,$$

for some positive constants d_0, d_1, β, where β measures the smoothness of the function. A density function f is termed super smooth, if its Fourier transform $\phi(t)$ decreases

exponentially, i.e. if

$$d_0|t|^{\beta_0}\exp\left(-\frac{|t|^\beta}{\gamma}\right) \;\le\; |\phi(t)| \;\le\; d_1|t|^{\beta_1}\exp\left(-\frac{|t|^\beta}{\gamma}\right) \qquad \text{as } \; t\to\infty,$$

for some positive constants d_0, d_1, β, γ and constants β_0, β_1. Examples of smooth distributions include the Gamma and the Laplace distribution. Super smooth distributions encompass stable distributions [8], which include the Normal and Cauchy distribution.

From the perspective of deconvolution, smooth and super smooth convolving distributions are numerically particularly challenging. Since the Fourier transform of the convolving distribution appears in the denominator of the inversion operator, its polynomial or exponential decay forces division by very small values at high frequencies. This, in turn, can lead to a strong amplification of numerical inaccuracies and, consequently, substantially restrict the performance of deconvolution procedures.

To mitigate this challenge, nonparametric deconvolution methods typically employ smoothing techniques to stabilize the estimation of $\widehat{\phi}_Y(t)$. A common approach is the use of a suitable kernel function with an appropriate bandwidth selection [1, 3, 2, 6]. While the kernel primarily determines the smoothing behavior and has a decisive influence on the stability of the estimator, the bandwidth acts as a supporting smoothing parameter, functioning as a cut-off for the numerically unstable parts of ϕ_Z. By carefully tuning these components, it is possible to reduce the amplification of noise while retaining essential structural information in the estimated density. A more detailed discussion of these techniques along with their implications for the stability of deconvolution estimators can be found in Section S2.

S1.2 Variance ratio between target and convolving distribution

If $\left|\widehat{\phi}_Z(t)\right|$ decays much more rapidly than $\left|\widehat{\phi}_X(t)\right|$, the numerical errors in the division might only occur in regions of $|t|$ in which the estimate $\widehat{\phi}_Y(t)$ of the target Fourier transform is already close to zero. In such cases, a solution to exclude the numerically unstable frequency components is to set the values in the erroneous regions to zero, which provides a good approximation for $\phi_Y(t)$. However, it is often the case that $\left|\widehat{\phi}_Z(t)\right|$ decays only slightly faster than $\left|\widehat{\phi}_X(t)\right|$, leading to considerable errors in regions of $|t|$ in which $\left|\widehat{\phi}_Y(t)\right|$ is not close to zero. Thus, in general, simply truncating the erroneous regions is not a viable solution.

In addition to the smoothness of $\widehat{\phi}_X$ and $\widehat{\phi}_Z$, the variance of the involved distributions plays a crucial role in the numerical robustness of the deconvolution. Specifically, another source of instability for the estimation $\widehat{\phi}_Y$ arises when the variance of the convolving variable X is relatively small compared to that of Y. To better understand this effect, we consider the following properties of convolved densities.

Let X and Y be two random variables, and let Z be a random variable with density $f_Z = f_Y * f_X$. Note that this does not necessarily imply that the distribution of Z coincides with the distribution of $Y + X$, particularly if X and Y are dependent. However, using the concept of independent copies [9], we can consider an i.i.d. copy $X_1 \sim X$ and an i.i.d. copy $Y_1 \sim Y$, such that X_1 and Y_1 are independent of each other and also independent of X and Y. It then follows that

$$f_Z = f_Y * f_X = f_{Y_1} * f_{X_1} = f_{Y_1+X_1},$$

which implies that $Z \stackrel{d}{=} Y_1 + X_1$. From this representation, the expectation and variance of Z is given by

$$\mathrm{E}(Z) = \mathrm{E}(Y) + \mathrm{E}(X) \quad \text{and} \quad \mathrm{Var}(Z) = \mathrm{Var}(Y) + \mathrm{Var}(X),$$

since $\mathrm{E}(X_1) = \mathrm{E}(X)$, $\mathrm{E}(Y_1) = \mathrm{E}(Y)$, and likewise for the variances.

This relationship also reveals a necessary condition for the applicability of deconvolution methods: Since the variance of the mixture Z equals the sum of the variances of X and Y, a meaningful deconvolution is only possible if $\mathrm{Var}(Z) > \mathrm{Var}(X)$. Otherwise, the variance attributed to the target variable Y would be negligible or even negative, making the estimation of f_Y infeasible or meaningless.

As the variance of a distribution increases, the Fourier transform of the corresponding density function decays more rapidly to zero. This is due to the property of Fourier transforms that for two random variables X_1 and X_2 for which $aX_1 \stackrel{\mathrm{d}}{=} X_2$ with a constant $a > 0$, the Fourier transform of X_2 is given by $\phi_{X_2}(t) = \phi_{X_1}(at)$. This indicates that for $a < 1$, the Fourier transform of f_{X_2} decays more slowly to zero than that of f_{X_1}, and for $a > 1$, ϕ_{X_2} decreases more rapidly to zero than ϕ_{X_1}. Furthermore, it follows from equation (9) of the main manuscript that the variance σ_X^2 of X is approximately equal to the variance σ_Z^2 of Z when σ_X^2 is much larger than the variance σ_Y^2 of Y, since in this case $\sigma_X^2 \approx \sigma_Y^2 + \sigma_X^2 = \sigma_Z^2$. In this situation, the Fourier transform $|\phi_Z(t)|$ will thus decay only slightly faster (if at all) to zero than the Fourier transform $|\phi_X(t)|$, if f_Z is similarly smooth as f_X, since the behavior of a Fourier transform is also determined by the smoothness of the density function. However, if f_Z is notably less smooth than f_X, the effect of $|\phi_Z(t)|$ decaying more slowly to zero is further amplified by the variance difference of X and Y.

In summary, if there exists a large difference in variances between the mixture variable Z and the target variable Y, this leads to a situation in which the deconvolution becomes highly sensitive to potential noise in the estimation of the corresponding Fourier transforms. This is analogous to the problems faced with (super) smooth convolving densities, as both scenarios involve rapidly decaying Fourier transforms that cause instability in the deconvolution process. We discuss in the following how these difficulties inherent in deconvolution are addressed by existing deconvolution procedures.

S2 Established Deconvolution Methods

S2.1 Measurement error deconvolution based on known error distribution

Wang and Wang [2] present a procedure for density estimation in additive measurement error models based on deconvolution kernel methods (DKM) and describe an implementation of this procedure in the R package `decon`. Like all the deconvolution procedures discussed in this section, this method is fundamentally based on Fourier transformation. The authors aim to estimate the true density of noisy data in an additive error model $Z_j = Y_j + X_j$, $j = 1, \ldots, n$, as shown in equation (1) of the main article and follow the idea discussed in Section "General idea of deconvolving densities", where the Fourier transform ϕ_Y of the target distribution is estimated using equation (4) of the main manuscript. For the measurement error variables $X_1, \ldots, X_n$, a symmetric and centered distribution is assumed. For this purpose, Wang and Wang [2] consider either normal or Laplace distributions. To estimate ϕ_Z based on the given contaminated data, the function

$$\widehat{\phi}_Z^{\mathrm{DKM}}(t) \;=\; \int e^{itx} \widehat{f}_Z^{\mathrm{DKM}}(x)\, dx$$

is proposed, where $\widehat{f}_Z^{\mathrm{DKM}}(x)$ is the ordinary kernel density estimator of f_Z given by

$$\widehat{f}_Z^{\mathrm{DKM}}(z) \;=\; \frac{1}{nh} \sum_{i=1}^{n} K_{\mathrm{DKM}} \left(\frac{z - Z_i}{h_{\mathrm{DKM}}} \right)$$

with $h_{\mathrm{DKM}} = h_{\mathrm{DKM}}(n) > 0$ being the bandwidth calculated by a simple rule of thumb [7], a plug-in method [10], or bootstrap methods [11]. The kernel function K_{DKM} is chosen so that it is suitable for the considered error distribution, i.e. for either the normal or the Laplace distribution. Using the deconvolution kernel method, the estimator for f_Y based on $\widehat{\phi}_Z^{\mathrm{DKM}}$ is given by the estimator

$$\widehat{f}_Y^{\mathrm{DKM}}(y) \;=\; \frac{1}{nh} \sum_{i=1}^{n} L_{\mathrm{DKM}} \left(\frac{y - Z_i}{h_{\mathrm{DKM}}} \right)$$

proposed by Stefanski and Carroll [10], where

$$L_{\mathrm{DKM}}(x) \;=\; \frac{1}{2\pi} \int e^{-itx} \frac{\widehat{\phi}_{K_{\mathrm{DKM}}}(t)}{\phi_X(t/h_{\mathrm{DKM}})}\, dt$$

is a kernel function with $\phi_{K_{\mathrm{DKM}}}(t)$ being the Fourier transform of the kernel K_{DKM}.

S2.2 Measurement error deconvolution with replicated data

Another challenge in deconvolving density functions in additive measurement error models arises when no assumption is made about the exact form of the error distribution, but replicated data of the contaminated observations are available. Delaigle et al. [12] developed a method, here, referred to as repeated measurement deconvolution (RMD), to address this particular deconvolution challenge. The authors consider error model (2) of the main article with the distinction that they allow the number of repeated measurements to differ between the different observations. Thus, they consider the model

$$Z_{jl} = Y_j + X_{jl}, \qquad l = 1, \ldots, n_j, \quad j = 1, \ldots, n$$

where the variables Y_j follow the target density f_Y, the random variables $X_{j1}, \ldots, X_{jn_j}$ are identically distributed with density f_X, and Y_j and X_{jl} are mutually independent.

To perform their deconvolution procedure, Delaigle et al. [12] estimate the Fourier transform $\phi_X^{\mathrm{RMD}}(t)$ of the error distribution using the available replicates under the assumption that the error distribution is symmetric and centered. More specifically, this Fourier transform is estimated by

$$\widehat{\phi}_X^{\mathrm{RMD}}(t) = \left| \frac{1}{R} \sum_{j=1}^{n} \sum_{(l_1,l_2)\in S_j} \cos\big(t\big(Z_{jl_1} - Z_{jl_2}\big)\big) \right|^{1/2},$$

where S_j is the set of all distinct pairs (l_1, l_2) with $1 \leq l_1 < l_2 \leq n_j$ of replicates for the j-th observation and R is the total number of distinct pairs, i.e. $R = \sum_{j=1}^{n} n_j(n_j - 1)/2$.

The estimator of f_Y is determined by

$$\hat{f}_Y^{\text{RMD}}(y) = \frac{1}{Mh_{\text{RMD}}} \sum_{j=1}^{n} w_j \sum_{l=1}^{n_j} \hat{L}_{\text{RMD}} \left(\frac{y - Z_{jl}}{h_{\text{RMD}}} \right),$$

where $M = \sum_j n_j$, the weights w_j are nonnegative and satisfy $\sum_j w_j n_j = M$, and

$$\hat{L}_{\text{RMD}}(x) = \frac{1}{2\pi} \int e^{-itx} \frac{\phi_K^{\text{RMD}}(t)}{\widehat{\phi}_X^{\text{RMD}}(t/h_{\text{RMD}}) + \rho} \, dt.$$

Here, ϕ_K^{RMD} denotes the compactly supported Fourier transform of a symmetric kernel function K_{RMD}, $h_{\text{RMD}} > 0$ is the bandwidth parameter, and $\rho \geq 0$ is a ridge parameter introduced to stabilize the denominator of the integral.

In the simulation study presented in Section "Deconvolution in additive measurement error models with replicated data" of the main article, we consider simulated data consisting of two replicated measurements for each observation so that $n_j = 2$, $j = 1, \ldots, n$. In this setting, we follow the construction suggested in the RMD approach in which the error distribution is estimated by calculating the differences between the two paired values z_{j1} and z_{j2}, $j = 1, \ldots, n$, from the mixed distribution. These differences are then scaled by $1/\sqrt{2}$, resulting in estimated values for the error distribution given by

$$\widehat{x}_j = \frac{z_{j1} - z_{j2}}{\sqrt{2}}, \quad j = 1, \ldots, n. \tag{S1}$$

This approach can be justified by the assumption made in the additive measurement error model. Since $Z_{j1} = Y_j + X_{j1}$ and $Z_{j2} = Y_j + X_{j2}$, it follows that $Z_{j1} - Z_{j2} = X_{j1} - X_{j2}$, $j = 1, \ldots, n$. Assuming the error distribution is centered, $\widehat{X}_j$ follows a distribution derived from the linear combination of two random variables following the error distribution that has the same expected value and variance as the error distribution. However, although the linear combination of two random variables with the same distribution is not necessarily of the same type as the original distribution, $\widehat{X}_j$ represents a promising approximation for a variable that follows the error distribution.

In the general case in which each observation Y_j is associated with $L \geq 2$ replicated measurements, i.e., $n_j = L$ for all $j = 1, \ldots, n$, the error distribution f_X can be approximated using a single linear combination of the measurements that eliminates the signal Y_j and preserves the variance of the error. Specifically, $\widehat{X}_j$ can be specified by

$$\widehat{X}_j = \frac{Z_{j1} - \frac{1}{L-1} \sum_{l=2}^{L} Z_{jl}}{\sqrt{1 + \frac{1}{L-1}}} = \frac{X_{j1} - \frac{1}{L-1} \sum_{l=2}^{L} X_{jl}}{\sqrt{1 + \frac{1}{L-1}}}, \quad j = 1, \ldots, n.$$

Under the assumption that the error distribution is centered, $\widehat{X}_j$ follows a distribution that results from a linear combination of L random variables following the error distribution and has the same expected value and variance as the error distribution.

S2.3 Deconvolution when data from the convolving density are available

Diggle and Hall [1] addressed the deconvolution problem, in which, in addition to data $z_1, \ldots, z_{n_z}$ from the mixed distribution, data $x_1, \ldots, x_{n_x}$ from the convolving distribution

are also available. It is important to note that these samples do not need to be paired. So the sizes of the given samples do not need to be identical. First, the Fourier transforms of the two distributions are estimated using the empirical Fourier transforms. Thus, the estimate of the Fourier transform ϕ_Z for the mixed distribution is given by

$$\widehat{\phi}_Z^{\text{emp}}(t) = \frac{1}{n_z} \sum_{j=1}^{n_z} e^{itz_j}. \tag{S2}$$

Analogously, the Fourier transform ϕ_X of the convolving distribution is estimated based on $x_1, \ldots, x_{n_x}$. To address the issues discussed in Section S1, Diggle and Hall [1] introduce a damping function d_{FDD}, which is an even, unimodal function with the properties that $d_{\text{FDD}}(0) = 1$ and $d_{\text{FDD}}(x) \to 0$ as $|x| \to \infty$. Based on this damping approach, we refer to their method as Fourier deconvolution with damping (FDD). Using this damping function factor, the estimate for the target density f_Y is given by

$$\widehat{f}_Y^{\text{FDD}}(y) = \frac{1}{2\pi} \int d_{\text{FDD}}(t) \frac{\widehat{\phi}_Z^{\text{emp}}(t)}{\widehat{\phi}_X^{\text{emp}}(t)} e^{-ity}\, dt.$$

An example of the damping function introduced by Bartlett [13] is given by

$$d_{\text{FDD}}(x) = \begin{cases} 1 - \frac{|x|}{M_{\text{FDD}}}, & \text{if } |x| \le M_{\text{FDD}} \\ 0, & \text{if } |x| > M_{\text{FDD}} \end{cases}.$$

This particular function is considered by Diggle and Hall [1] in their simulation study, in which they estimate M_{FDD} by $\widehat{M}_{\text{FDD}} = \widehat{p}/\sqrt{2}$, where $\widehat{p}$ is generated using a least squares fit of $\log\bigl|\widehat{\phi}_X^{\text{emp}}(t)\bigr|$ against $\log|t|$ over values of t for which the log–log relation is approximately linear.

S2.4 Deconvolution using data from the convolving distribution from a second experiment

Another deconvolution procedure for situations in which data for both the mixed and the convolving density are available was introduced by Neumann [3]. This author addressed the deconvolution problem in such situations by deriving minimax convergence rates for deconvolution (MCD) of the density estimator, quantifying the fastest possible rate at which the estimation error can decrease uniformly over a class of target densities, even in situations in which f_X is unknown but samples from the convolving distribution are available. Neumann [3] motivated the topic of deconvolution by suggesting that data from the convolving density could arise from a second, independent experiment. The author proposes to estimate the target density f_Y by

$$\widehat{f}_Y^{\text{MCD}}(y) = \frac{1}{2\pi} \int K_{\text{MCD}} \left(\frac{t}{h_n^{\text{MCD}}} \right) \widehat{\phi}_Z^{\text{emp}}(t) e^{-iyt} I \left(\left| \widehat{\phi}_X(t) \right| \ge n_x^{-1/2} \right) dt,$$

where K_{MCD} is a kernel function, h_n^{MCD} is the bandwidth for this kernel function that depends on the mixed distribution, and the indicator function suppresses fluctuations in the critical region. Neumann [3] emphasizes that, from a practical standpoint, the principal advantage of this estimator over other established estimation methods is the ability to use the same bandwidth in scenarios involving unknown error distributions as in situations in which the error distribution is known. This uniformity in bandwidth

selection simplifies the estimation process and enhances the applicability of the estimator across different contexts without compromising accuracy.

S3 Additional Methodological Details

S3.1 Estimation of the Fourier transforms

As first discussed in Section "Methods" of the main paper, it is common practice to address the issue of highly oscillating tails of $\widehat{\phi_Y}$ by estimating the Fourier transforms based on the given data $x_1, \ldots, x_{n_x}$ and $z_1, \ldots, z_{n_z}$ using a smoothing kernel or by determining the empirical Fourier transforms.

In NPFD, however, we follow another approach, in which we first estimate the density functions f_X and f_Z based on the given data for X and Z, respectively, and then, numerically integrate according to the definition of Fourier transforms. The underlying idea is to initially generate smooth estimates $\widehat{f_X}$ and $\widehat{f_Z}$ for these density functions based on the observed data, and afterwards use an appropriate numerical integration method to achieve precise estimates for the Fourier transforms ϕ_X and ϕ_Z that exhibit only minimal fluctuations in the tails.

In the following, we describe in the following the procedure for estimating f_X, where we set $n = n_x$. Analogously, f_Z can be estimated using $z_1, \ldots, z_n$. For the estimation of the density f_X, a procedure proposed by Efron and Tibshirani [14] with modifications suggested by Schwender and Ickstadt [15] is employed. This density estimation method involves a combination of histogram construction, natural cubic splines, and Poisson regression. It is designed to achieve a precise and robust density estimation.

In this density estimation, first, the number of intervals that should be used in the histogram is determined. There are several methods for selecting the optimal number of intervals, each with its own advantages. E.g., the method of Scott [16] minimizes the mean integrated squared error (MISE) [17] between the estimated density and the true density, providing a statistically grounded approach that adjusts the bin width based on the variability of the data. The Wand [18] method, which is used in NPFD, is also based on minimizing the MISE to select the bin width. In contrast to the procedure of Scott [16], this approach makes use of kernel density estimation, creating a precise histogram representation by flexibly adapting to the distribution of the data.

Once the number n_I of intervals is determined, a histogram of the observations $x_1, \ldots, x_n$ is created. The range of $x_1, \ldots, x_n$ is divided into n_I equally-spaced, distinct intervals $I_1, \ldots, I_{n_I}$ with interval boundaries $b_1, \ldots, b_{n_I+1}$. The midpoints m_i of the intervals and the number c_i of observations falling into the interval I_i are then

$$m_i = \frac{b_i + b_{i+1}}{2} \qquad \text{and} \qquad c_i = \sum_{j=1}^{n} I(x_j \in I_i), \qquad i = 1, \ldots, n_I.$$

Next, a natural cubic spline with a basis vector $\boldsymbol{S}^\top(m_i) = \left[S_1(m_i) \ldots S_{k+2}(m_i)\right]$, $i = 1, \ldots, n_I$, is constructed from the midpoints of the histogram intervals. The vector $\boldsymbol{S}(m_i)$ consists of the values of the basis functions $S_1, \ldots, S_{k+2}$ evaluated at m_i. These splines are constructed to smooth the estimation of the density function and to be robust against outliers. The number J of knots of the natural cubic spline is specified by the selected degrees of freedom of this spline, since these degrees of freedom are given by $J + 1$. The positions of the knots are determined either based on the mode or the median of the data, where in NPFD five degrees of freedom and the mode are used as standard setting (that, however, can be changed depending on the considered data).

When the mode is used, the knots of the spline are determined by quantiles of $x_1, \ldots, x_n$ that are chosen relative to the mode. To accomplish this, first, the mode J_{mod} is calculated by the midpoint of the interval with the highest count of data points, i.e. by $J_{\text{mod}} = m_{\arg\max(c_1,\ldots,c_{n_I})}$. Afterwards, the proportion r of the interval midpoints that are smaller than or equal to the interval midpoint of the mode is determined. Thus, this proportion is given by

$$r = \frac{1}{n_I} \sum_{i=1}^{n_I} I(m_i \leq J_{\text{mod}}).$$

Using the normalizing factor $d = (J+1)/2$, the first set of quantiles to the left of the mode J_{mod} is determined by equidistant values between 0 and r, i.e. by

$$q_i = \frac{i \cdot r}{d}, \qquad i = 1, 2, \ldots, \left\lfloor \frac{J}{2} \right\rfloor.$$

The second set of quantiles to the right of J_{mod} is given by equidistant values between r and 1, i.e. by

$$q_j = 1 - \frac{(1-r)\cdot(J-j+1)}{d}, \qquad j = \left\lceil \frac{J}{2} \right\rceil, \left\lceil \frac{J}{2} \right\rceil + 1, \ldots, J.$$

This process ensures that the knots for the natural cubic spline are positioned in a manner that accurately reflects the distribution of the data, particularly around the mode.

The knots are essential for defining the piecewise polynomial segments of the natural cubic spline. Proper placement of these knots ensures that the splines can adapt smoothly to the data, capturing its underlying structure without overfitting. By placing knots at specific quantiles, the splines are better positioned to represent the distribution of the data. This is especially important in regions with higher data density such as around the mode or median, ensuring that the splines capture the important features of the data. The number and placement of knots determine the flexibility of the spline. More knots allow the spline to fit the data more closely, while fewer knots result in a smoother spline. By adjusting the number of knots based on quantiles, the method balances between overfitting and underfitting, providing a robust density estimation.

For the density estimation, a Poisson regression model is fitted using the basis functions $S_1, \ldots, S_{k+2}$ as explanatory variables and the counts from the histogram as the outcome variable. The likelihood function for the Poisson regression is given by

$$L(\boldsymbol{\beta}) = \prod_{i=1}^{n_I} \frac{e^{-\lambda_i} \lambda_i^{c_i}}{c_i!},$$

where λ_i is the expected count in the i-th interval, $i = 1, \ldots, n_I$. This expected count is modeled as

$$\lambda_i = \exp\big(\boldsymbol{S}^\top(m_i)\boldsymbol{\beta}\big), \qquad i = 1, \ldots, n_I,$$

where the vector $\boldsymbol{\beta} \in \mathbb{R}^{k+2}$ contains the regression parameters belonging to the basis functions of the natural cubic spline. Fitting the Poisson regression model leads to the fitted values

$$\widehat{c}_i = \exp\big(\boldsymbol{S}^\top(m_i)\widehat{\boldsymbol{\beta}}\big), \qquad i = 1, \ldots, n_I.$$

To obtain the density estimates at the data points $x_1, \ldots, x_n$, the values of the basis functions are evaluated at each x_i to compute the unscaled density estimates as $\exp\big(\boldsymbol{S}^\top(x_i)\widehat{\boldsymbol{\beta}}\big)$,

$i = 1, \ldots, n$. The fitted counts are then scaled to obtain values

$$\widehat{f}_{X,i} \;=\; \frac{\exp\big(\boldsymbol{S}^\top(x_i)\widehat{\boldsymbol{\beta}}\big)}{\Delta \cdot \sum_{i=1}^{n_I} c_i}, \qquad i = 1, \ldots, n,$$

where Δ represents the equal width of all n_I intervals. To numerically integrate the estimated densities with the highest possible accuracy, we use the continuous form of the density estimation, which is given for any x in the range of $x_1, \ldots, x_n$ by

$$\widehat{f}_X(x) = \frac{\exp\big(\boldsymbol{S}^\top(x)\widehat{\boldsymbol{\beta}}\big)}{\Delta \cdot \sum_{i=1}^{n_I} c_i}, \tag{S3}$$

where $\boldsymbol{S}^\top(x)$ is the continuous form of the considered vector of basis functions of the natural cubic spline. Using (S3), values of the estimated density can be determined at any arbitrary point within the range of the estimated function. For values of x outside of the range of the observations $x_1, \ldots, x_n$, the estimate $\widehat{f}_X(x)$ is set to 0.

S3.2 Estimation of the Fourier transforms

In Section "Simulation study" of the main manuscript and Section S4 of this supplementary file, we consider simulation scenarios with sufficient numbers of observations to estimate the densities f_X and f_Z using the described procedure. Additionally, we consider situations in which the sample sizes are very small, making reliable density estimation no longer feasible. In these situations, we resort to the direct estimation of the Fourier transform by determining the empirical Fourier transform as described in (S2). This approach also yields promising results for larger sample sizes, but the estimates of the target density are often less smooth compared to the ones obtained by first estimating the densities f_X and f_Z.

After having estimated f_X and f_Z, the Fourier transforms of $\widehat{f}_X$ and $\widehat{f}_Z$ have to be estimated. For this, numerical integration is required, where in NPFD the rectangle rule [19] is employed. For a function $f(s,t) : \mathbb{R}^2 \to \mathbb{R}$, define the integral

$$F_{\mathcal{I}}(t) \;=\; \int_u^v f(s,t)\, ds$$

that should be computed over the interval $\mathcal{I} = [u, v]$. To perform the numerical integration, we proceed as follows. First, ℓ equidistant points $s_j,\ j = 1, \ldots, \ell$, covering the interval $\mathcal{I}$ are chosen. Next, the function $f(s,t)$ at each of these equidistant points s_j is evaluated in s. The integral $F_{\mathcal{I}}$ is then estimated by taking the average of the function values and multiplying by the length $v - u$ of the interval $\mathcal{I}$ using

$$\widehat{F}_{\mathcal{I}}(t) = \frac{v-u}{\ell} \sum_{j=1}^{\ell} f(s_j, t).$$

To apply numerical integration for estimating the Fourier transform of $\widehat{f}_X$, denote the function $f(s,t)$ as $\widehat{f}_X(s)\exp(ist)$. The estimate of the Fourier transform over the interval $\mathcal{I} = [u, v]$ is then computed as

$$\widehat{\phi}_X(t) = \frac{v-u}{\ell} \sum_{j=1}^{\ell} \widehat{f}_X(s_j)\exp(is_j t),$$

where we introduce the procedure for the choice of the interval range in Section S3.4

A major advantage of numerical integration via the rectangle rule lies in its remarkable flexibility, allowing it to be applied to arbitrary integration domains, even if they are complex and irregular. This versatility can be highly beneficial in the Fourier inversion step in NPFD, when we aim to numerically integrate the estimated Fourier transform of the target distribution f_Y. Moreover, the error associated with numerical integration using the rectangle rule can be accurately estimated and is proportional to $1/\ell^2$ [19]. Consequently, increasing ℓ systematically reduces the error, providing a straightforward method to enhance the precision of the integration.

S3.3 Choice of the truncation threshold ε

In the vast majority of simulation settings considered in the main manuscript, a default value of $\varepsilon = 0.001$ was used unless stated otherwise. This value was found to perform reliably across a wide range of scenarios in extensive preliminary experiments. Since this default value is very small, it retains almost the entire estimated Fourier transform for the inversion step, while still providing effective stabilization of tail behavior.

In certain situations, e.g., for very small sample sizes or specific distributional configurations (e.g., pronounced local features such as sharp peaks or multiple modes), the condition

$$\left|\left(\widehat{\phi}_{\tilde{Y}}(t)\right)^N\right| < \varepsilon$$

may only be satisfied for comparatively large values of N, in which case the induced smoothing can become overly strong and may substantially attenuate such local structure in $\widehat{f}_Y^{\mathrm{NPFD}}$. As discussed in Section "N-Power Fourier Deconvolution" of the main manuscript and visualized in Section S4.1, excessively large values of N can induce oversmoothing. In such cases, a less stringent truncation, i.e., a larger value of ε, can be preferable in order to avoid unnecessary loss of structural detail.

Since the appropriate balance between stabilization and resolution depends on several factors, including sample size, variance ratios, and the underlying distributional setting, a universally optimal choice of ε cannot be derived from theoretical considerations.

As a practical diagnostic, the plausibility of a chosen value for ε might be assessed by convolving the NPFD estimate $\widehat{f}_Y^{\mathrm{NPFD}}$ with an estimate of the convolving density f_X and comparing the resulting density to an estimate of f_Z. Large discrepancies indicate that the selected power N (and thus the induced truncation) may be excessive, in which case revisiting the truncation threshold ε can be advisable. This diagnostic is not used to optimize estimation accuracy and does not involve comparison to the true target density, which makes it feasible in real-data settings, but serves to assess consistency with the assumed convolution model.

S3.4 The choice of N

The following algorithm specifies how the power N is chosen in the NPFD procedure.

Algorithm S1 Algorithm to choose the power N

Input:

$\boldsymbol{x}, \boldsymbol{z}$ – Data vectors
$N_{\max}$ – Maximum value for N
K – Number of equidistant Fourier transform points
t_K – Upper bound of the Fourier transform points
ε – Small threshold value
δ – Small margin constant

Output: N – Chosen power

1: **for** $N \in \{1, \dots, N_{\max}\}$ **do**
2: $\quad a = \frac{1}{\sqrt{N}}$
3: $\quad b_{\boldsymbol{x}} = \left(\frac{1}{N} - \frac{1}{\sqrt{N}}\right) \cdot \bar{\boldsymbol{x}}$
4: $\quad b_{\boldsymbol{z}} = \left(\frac{1}{N} - \frac{1}{\sqrt{N}}\right) \cdot \bar{\boldsymbol{z}}$
5: $\quad \tilde{\boldsymbol{x}} = a \cdot \boldsymbol{x} + b_{\boldsymbol{x}}$
6: $\quad \tilde{\boldsymbol{z}} = a \cdot \boldsymbol{z} + b_{\boldsymbol{z}}$
7: $\quad$ Compute densities $\widehat{f}_{\tilde{X}}$ and $\widehat{f}_{\tilde{Z}}$
8: $\quad$ **for** $k \in \left\{\frac{K+1}{2}, \dots, K\right\}$ **do**
9: $\qquad$ Compute estimation $\widehat{\phi}_{\tilde{Y}}(t_k)$
10: $\qquad$ **if** $\left|\widehat{\phi}_{\tilde{Y}}(t_k)\right|^N > 1$ **then**
11: $\qquad\quad$ **break**
12: $\qquad$ **end if**
13: $\qquad$ **if** $\left|\widehat{\phi}_{\tilde{Y}}(t_k)\right|^N < \varepsilon$ **then**
14: $\qquad\quad$ Compute Fourier transform at $t_k + \delta$
15: $\qquad\quad$ **if** $\left|\widehat{\phi}_{\tilde{Y}}(t_k + \delta)\right|^N < \varepsilon$ **then**
16: $\qquad\qquad$ **go to step 23**
17: $\qquad\quad$ **else**
18: $\qquad\qquad$ **break**
19: $\qquad\quad$ **end if**
20: $\qquad$ **end if**
21: $\quad$ **end for**
22: **end for**
23: **return** N

S3.5 Asymptotical behavior of NPFD in N based on empirical Fourier transforms

In this section, we derive the asymptotic behavior of NPFD in N.

For sample vectors $\boldsymbol{x} = \left[x_1, \ldots, x_{n_x}\right]^\top$ and $\boldsymbol{z} = \left[z_1, \ldots, z_{n_z}\right]^\top$, consider the empirical Fourier transforms

$$\widehat{\phi}_X^{\text{emp}}(t) = \frac{1}{n_x}\sum_{j=1}^{n_x} e^{itx_j} \quad \text{and} \quad \widehat{\phi}_Z^{\text{emp}}(t) = \frac{1}{n_z}\sum_{j=1}^{n_z} e^{itz_j}.$$

Furthermore, for $N \in \mathbb{N}$, consider the linear scaling $a = 1/\sqrt{N}$ and the empirical shifts

$$b_x = \Big(\frac{1}{N} - \frac{1}{\sqrt{N}}\Big)\bar{\boldsymbol{x}}, \qquad b_z = \Big(\frac{1}{N} - \frac{1}{\sqrt{N}}\Big)\bar{\boldsymbol{z}}, \qquad b_y = b_z - b_x.$$

Setting $\tilde{x}_j = ax_j + b_x$ and $\tilde{z}_j = az_j + b_z$, define the corresponding empirical Fourier transforms

$$\widehat{\phi}_{\tilde{X}}^{\text{emp}}(t) = \frac{1}{n_x}\sum_{j=1}^{n_x} e^{it\tilde{x}_j} \quad \text{and} \quad \widehat{\phi}_{\tilde{Z}}^{\text{emp}}(t) = \frac{1}{n_z}\sum_{j=1}^{n_z} e^{it\tilde{z}_j}.$$

Then, we have

$$\widehat{\phi}_{\tilde{X}}^{\text{emp}}(t) = e^{itb_x}\widehat{\phi}_X^{\text{emp}}\Big(\frac{t}{\sqrt{N}}\Big) \quad \text{and} \quad \widehat{\phi}_{\tilde{Z}}^{\text{emp}}(t) = e^{itb_z}\widehat{\phi}_Z^{\text{emp}}\Big(\frac{t}{\sqrt{N}}\Big).$$

Hence, $\widehat{\phi}_Y^{\text{NPFD}}$ based on empirical Fourier transforms can be expressed as

$$\widehat{\phi}_Y^{\text{NPFD}}(t) = \left(\frac{\widehat{\phi}_{\tilde{Z}}^{\text{emp}}(t)}{\widehat{\phi}_{\tilde{X}}^{\text{emp}}(t)}\right)^N = e^{iN(b_z - b_x)t}\left(\frac{\widehat{\phi}_Z^{\text{emp}}(t/\sqrt{N})}{\widehat{\phi}_X^{\text{emp}}(t/\sqrt{N})}\right)^N. \tag{S4}$$

To study the limit $N \to \infty$ for fixed $t \in \mathbb{R}$, it suffices to control the empirical Fourier transforms in a neighborhood of the origin, since $s = t/\sqrt{N} \to 0$. Using the pointwise expansion

$$e^{isx_j} = 1 + isx_j - \frac{s^2x_j^2}{2} + o(s^2), \qquad s \to 0,$$

and averaging over $j = 1, \ldots, n_x$ yields

$$\widehat{\phi}_X^{\text{emp}}(s) = \frac{1}{n_x}\sum_{j=1}^{n_x} e^{isx_j} = 1 + is\,\bar{\boldsymbol{x}} - \frac{s^2\,\overline{\boldsymbol{x}^2}}{2} + o(s^2), \qquad s \to 0,$$

where $\overline{\boldsymbol{x}^2} = n_x^{-1}\sum_{j=1}^{n_x} x_j^2$. To use

$$\log(1+u) = u - \frac{u^2}{2} + o(u^2), \qquad u \to 0,$$

we write

$$\widehat{\phi}_X^{\text{emp}}(s) = 1 + is\,\bar{\boldsymbol{x}} - \frac{s^2\,\overline{\boldsymbol{x}^2}}{2} + o(s^2) = 1 + u(s), \qquad s \to 0,$$

where $u(s) = is\,\bar{\boldsymbol{x}} - \frac{s^2\,\overline{\boldsymbol{x}^2}}{2} + o(s^2) = O(s)$. Since

$$u(s)^2 = \big(is\,\bar{\boldsymbol{x}}\big)^2 + o(s^2) = -s^2\,\bar{\boldsymbol{x}}^{\,2} + o(s^2),$$

we have

$$\log \widehat{\phi}_X^{\text{emp}}(s) = is\,\bar{\boldsymbol{x}} - \frac{s^2\,\overline{\boldsymbol{x}^2}}{2} - \frac{-s^2\,\bar{\boldsymbol{x}}^{\,2}}{2} + o(s^2) = is\,\bar{\boldsymbol{x}} - \frac{s^2\widehat{\sigma}_X^2}{2} + o(s^2),$$

where $\widehat{\sigma}_X^2 = \overline{\boldsymbol{x}^2} - \bar{\boldsymbol{x}}^{\,2}$. Analogously,

$$\log \widehat{\phi}_Z^{\text{emp}}(s) = is\,\bar{\boldsymbol{z}} - \frac{s^2\,\widehat{\sigma}_Z^2}{2} + o(s^2), \qquad s \to 0,$$

where $\overline{\boldsymbol{z}^2} = n_z^{-1}\sum_{j=1}^{n_z} z_j^2$ and $\widehat{\sigma}_Z^2 = \overline{\boldsymbol{z}^2} - \bar{\boldsymbol{z}}^{\,2}$.

Substituting $s = t/\sqrt{N}$ and the difference of the logarithms by N yields

$$N\Big(\log \widehat{\phi}_Z^{\text{emp}}(t/\sqrt{N}) - \log \widehat{\phi}_X^{\text{emp}}(t/\sqrt{N})\Big) = it\sqrt{N}\big(\bar{\boldsymbol{z}} - \bar{\boldsymbol{x}}\big) - \frac{t^2\big(\widehat{\sigma}_Z^2 - \widehat{\sigma}_X^2\big)}{2} + o(1).$$

Moreover, by the specific choice of shifts b_x and b_z, it holds that

$$iN(b_z - b_x)t = it\big(\bar{\boldsymbol{z}} - \bar{\boldsymbol{x}}\big) - it\sqrt{N}\big(\bar{\boldsymbol{z}} - \bar{\boldsymbol{x}}\big).$$

Combining these equations with (S4) gives, for each fixed t,

$$\log \widehat{\phi}_Y^{\text{NPFD}}(t) = it\big(\bar{\boldsymbol{z}} - \bar{\boldsymbol{x}}\big) - \frac{t^2\big(\widehat{\sigma}_Z^2 - \widehat{\sigma}_X^2\big)}{2} + o(1), \qquad N \to \infty,$$

and therefore

$$\widehat{\phi}_Y^{\text{NPFD}}(t) \;\longrightarrow\; \exp\Big(it\,\widehat{\mu}_Y - \frac{\widehat{\sigma}_Y^2 t^2}{2}\Big),$$

which is the Fourier transform of the $N(\widehat{\mu}_Y, \widehat{\sigma}_Y^2)$ distribution with parameters

$$\widehat{\mu}_Y = \bar{\boldsymbol{z}} - \bar{\boldsymbol{x}} \quad \text{and} \quad \widehat{\sigma}_Y^2 = \widehat{\sigma}_Z^2 - \widehat{\sigma}_X^2.$$

This shows that, as $N \to \infty$, the NPFD estimator based on empirical Fourier transforms converges asymptotically to a normal density with mean $\widehat{\mu}_Y$ and variance $\widehat{\sigma}_Y^2$.

S4 Simulation Study

S4.1 Behavior of NPFD on the Fourier domain across power values

To evaluate the effect of the power N in NPFD, we considered an illustrative example of the behavior of the Fourier transform estimate $\widehat{\phi}_Y^{\mathrm{NPFD}}$ for different values of N. In this simulation example, the target density f_Y was the Gamma$(4,1)$ density and f_X was the density of the $N(0,0.5)$ distribution. For the application of NPFD based on density estimates of f_X and f_Z, we considered a sample size of $n = n_x = n_z = 500$, and for its application based on the empirical Fourier transforms, $n = n_x = n_z = 100$ was used as sample size. To visualize potential under- and oversmoothing arising from an inappropriate choice of N, we considered $N \in \{1,2,3,4,5,10,100,1000\}$.

In Figures S1 and S2, these representative results of these two applications of NPFD are shown. As expected, for $N = 1$ both variants yielded noticeably oscillatory estimates (Figures S1 and S2 (a)), a common behavior also observed for other Fourier-based deconvolution methods prior to smoothing (see Section "General idea of deconvolving densities" of the manuscript). The fluctuations are more pronounced for the smaller sample size, whereas they are relatively mild for $n = 500$. Besides the larger sample size, the density estimation based NPFD can further reduce fluctuations, since the preliminary density estimation provides additional smoothing of the observed data.

The stabilizing mechanism of NPFD is apparent in both figures. Although the tails of the estimates are unstable for $N = 1$, the corresponding values still lie in the range $[-1,1]$ usually for $t \in [-3,3]$ and are typically smaller than those near $t = 0$. Taking the N-th power, therefore, primarily attenuates tail fluctuations, while preserving the overall structure near the origin. This behavior is clearly visible for $N \geq 2$ (see Figures S1 and S2 (b)–(h)). For moderate values ($N \leq 5$), the estimated transforms mostly closely match the true Fourier transform ϕ_Y, while remaining smooth. For larger N, the transforms become increasingly stable but deviate more from ϕ_Y due to oversmoothing.

It is noteworthy that this illustrative example does not generally induce the goodness for the considered values of power N. Depending on the different distributions and the variance ratios involved in specific scenarios, smaller and especially larger values of the power N can be justifiable.

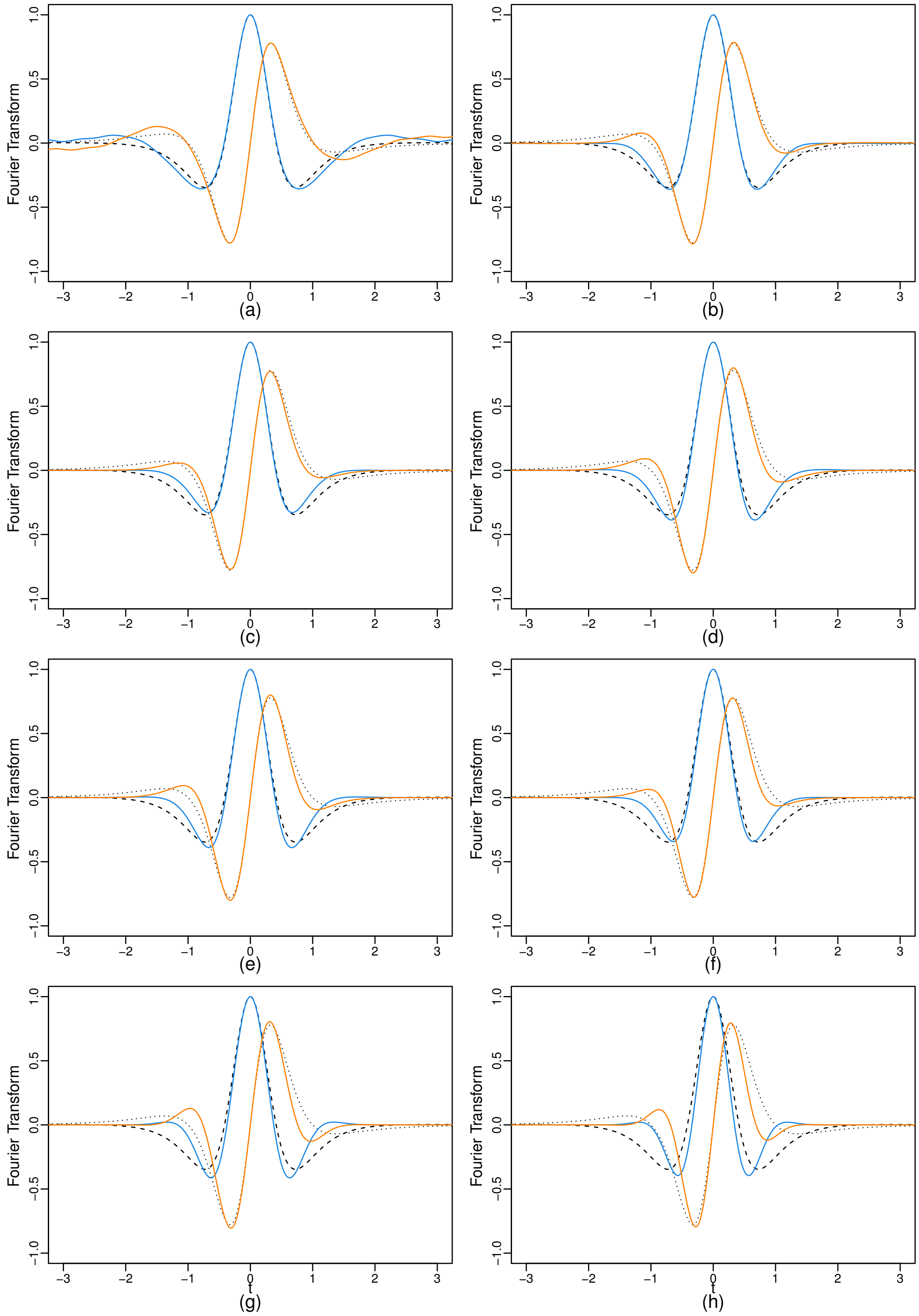

Figure S1: Estimated Fourier transforms $\widehat{\phi}_Y^{\mathrm{NPFD}}$ (blue: real part; orange: imaginary part) based on estimated densities with $n = 500$. The real (black; dashed) and imaginary (black; dotted) parts of the true transform ϕ_Y are shown for reference. Panels (a)-(h) correspond to $N = 1, 2, 3, 4, 5, 10, 100, 1000$, respectively.

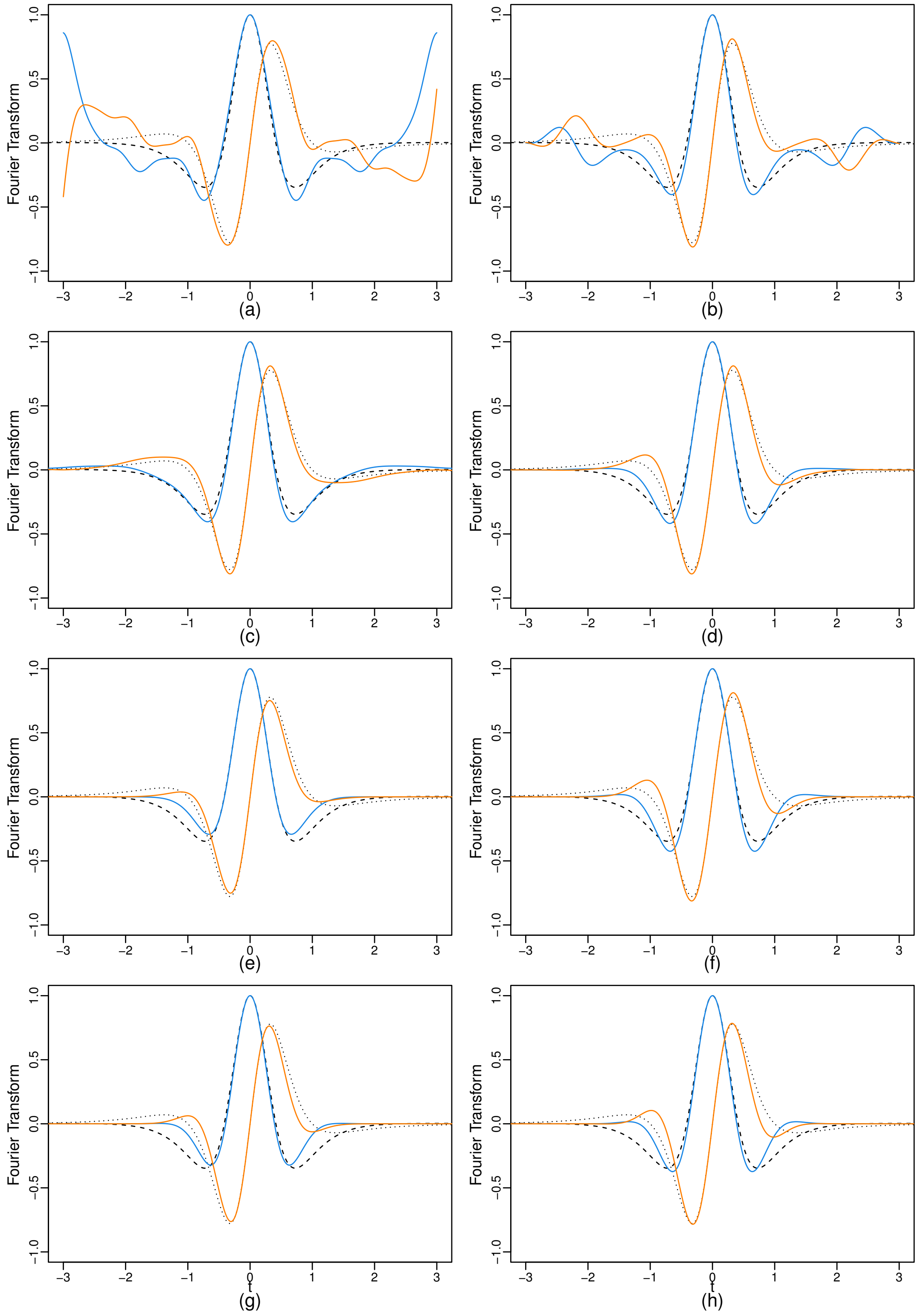

Figure S2: Estimated Fourier transforms $\widehat{\phi}_Y^{\mathrm{NPFD}}$ (blue: real part; orange: imaginary part) based on empirical Fourier transforms with $n = 100$. The real (black; dashed) and imaginary (black; dotted) parts of the true transform ϕ_Y are shown for reference. Panels (a)-(h) correspond to $N = 1, 2, 3, 4, 5, 10, 100, 1000$, respectively.

S4.2 Further results of the comparison of NPFD with FDD

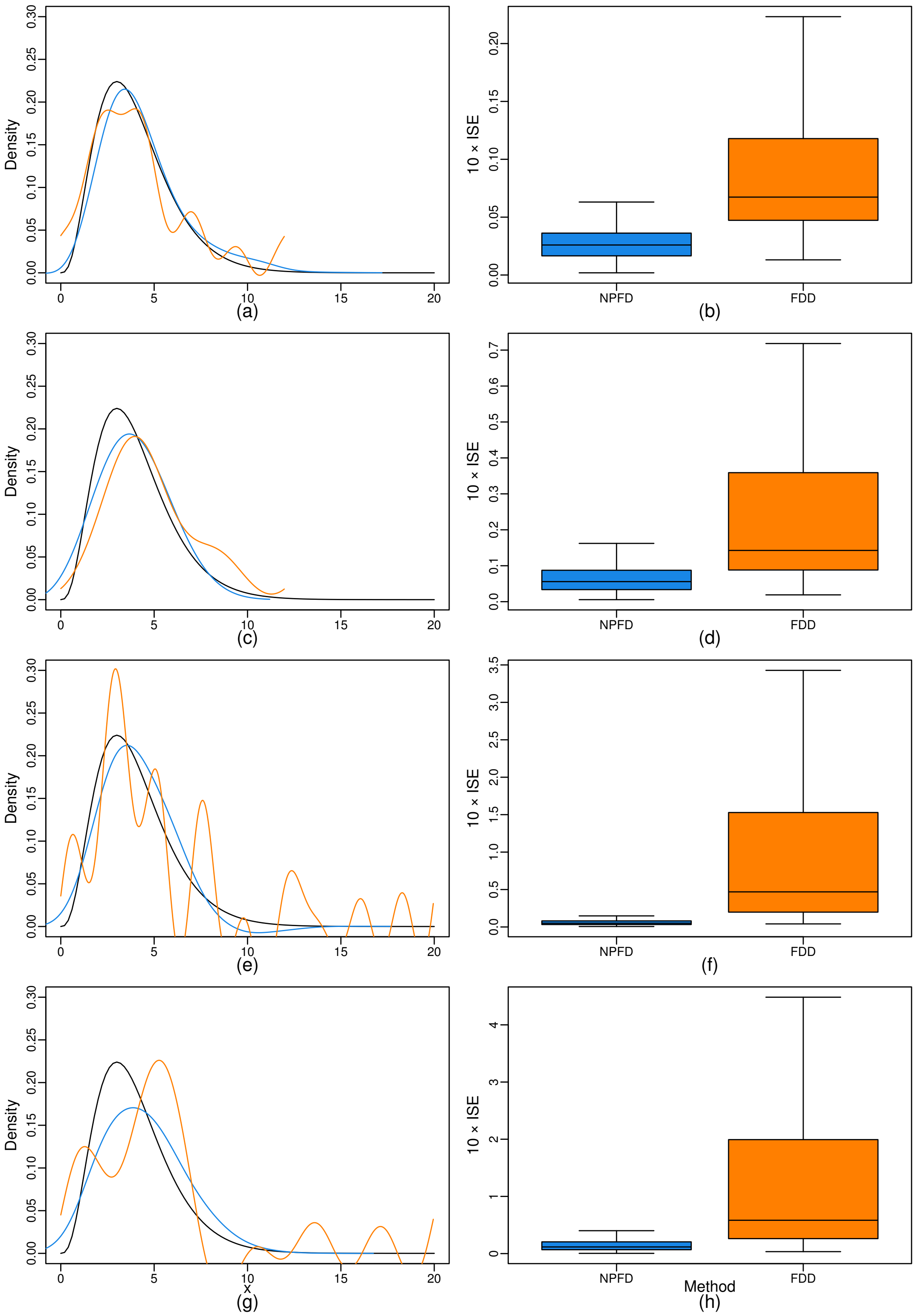

Figure S3: (a), (c), (e), (g): Comparison of a representative density estimate $\widehat{f}_Y^{\,\mathrm{NPFD}}$ (blue) to a representative density estimate $\widehat{f}_Y^{\,\mathrm{FDD}}$ (orange) with the true density f_Y (black) for Scenario 3 with sample sizes of 500 (a) and 100 (c), as well as for Scenario 4 with sample sizes of 500 (e) and 100 (g). (b), (d), (f), (h): Box plots of the values of $10 \times$ ISE of the density estimators (without outliers) in the corresponding 500 simulated data sets from (a), (c), (e), and (g), respectively.

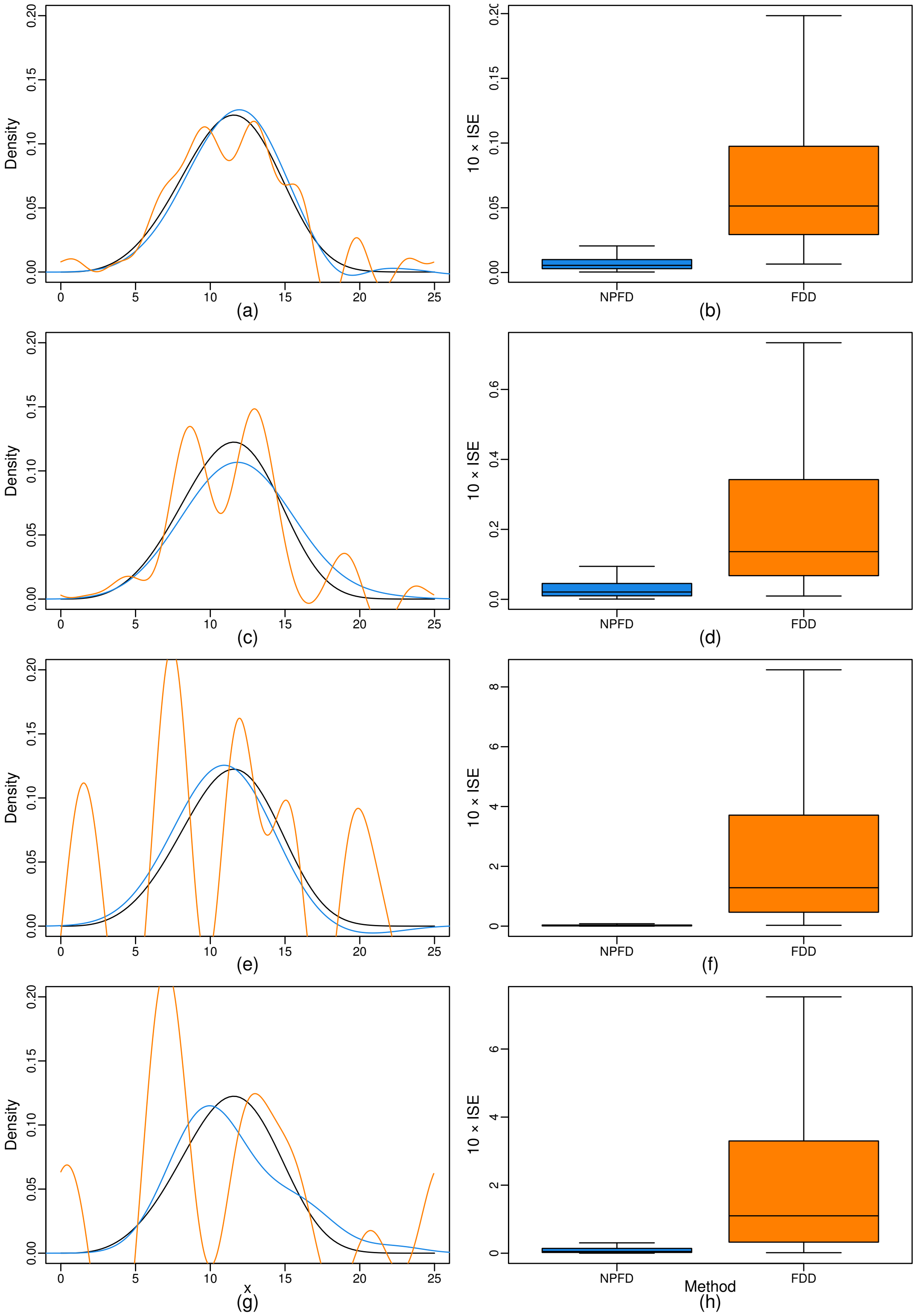

Figure S4: (a), (c), (e), (g): Comparison of a representative density estimate $\widehat{f}_Y^{\,\mathrm{NPFD}}$ (blue) to a representative density estimate $\widehat{f}_Y^{\,\mathrm{FDD}}$ (orange) with the true density f_Y (black) for Scenario 5 with sample sizes of 500 (a) and 100 (c), as well as for Scenario 6 with sample sizes of 500 (e) and 100 (g). (b), (d), (f), (h): Box plots of the values of $10 \times$ ISE of the density estimators (without outliers) in the corresponding 500 simulated data sets from (a), (c), (e), and (g), respectively.

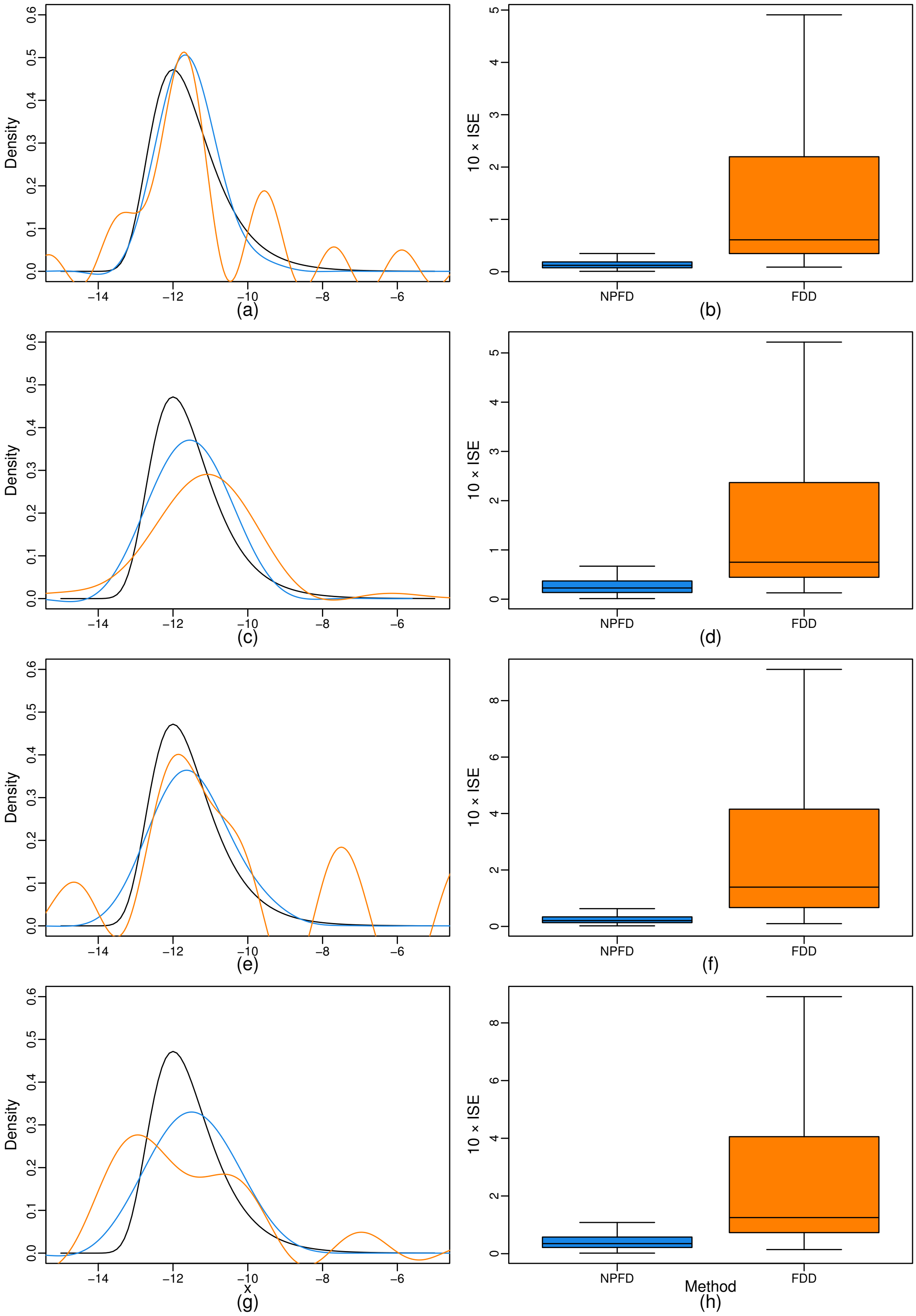

Figure S5: (a), (c), (e), (g): Comparison of a representative density estimate $\widehat{f}_Y^{\mathrm{NPFD}}$ (blue) to a representative density estimate $\widehat{f}_Y^{\mathrm{FDD}}$ (orange) with the true density f_Y (black) for Scenario 7 with sample sizes of 500 (a) and 100 (c), as well as for Scenario 8 with sample sizes of 500 (e) and 100 (g). (b), (d), (f), (h): Box plots of the values of $10 \times$ ISE of the density estimators (without outliers) in the corresponding 500 simulated data sets from (a), (c), (e), and (g), respectively.

In Figures S3-S5, the results from Scenarios 3-8 of the comparison from NPFD with FDD not displayed in the main article are presented. While the NPFD estimates become visually smoother as the sample size increases, Figures S3 and S5 (a), (c) reveal that, in some cases, the FDD estimates may exhibit increased oscillations with growing sample sizes. A reason for this might be that in each of these figures the estimates of a single representative replicate, selected as the data set whose $10 \times$ ISE is closest to the median across all simulation runs, is shown. As a consequence, individual realizations may occasionally exhibit visual differences across sample sizes, such as increased oscillations, even when the overall performance improves with larger sample sizes. Additionally, for larger sample sizes the empirical distribution of the convolving variable X in a given replicate may appear smoother than the empirical distribution in a replicate with smaller sample size. This can increase numerical sensitivity in the deconvolution step and, in turn, lead to more oscillatory FDD estimates for that replicate. A similar behavior can also be found in the article of Diggle and Hall [1], in which representative estimates based on larger sample sizes appear more accurate overall, while those based on smaller samples may look visually smoother despite being less accurate.

**Scenario**	n	N	**Scenario**	n	N
**1**	500	2	**5**	500	4
	100	3		100	5
**2**	500	2	**6**	500	4
	100	4		100	9
**3**	500	2	**7**	500	3
	100	4		100	12
**4**	500	3	**8**	500	4
	100	4		100	4

Table S1: Power values N used in the NPFD estimation for the representative estimate of each scenario in the comparison with FDD.

S4.3 Deconvolution in additive measurement error models with known error distribution

In order to evaluate how NPFD performs in applications to additive measurement error models with known error distributions, we considered two specific settings that were also considered by Wang and Wang [2] and compared the performance of NPFD with their proposed estimator $\widehat{f}_Y^{\mathrm{DKM}}$, presented in Section S2.1. In Scenario 1, f_Y was modeled as a standard normal distribution, while f_X followed a Laplace$(0, 0.5)$ distribution. In Scenario 2, the mixture $N(-3, 1) + N(3, 1)$ of two normal distributions was considered as distribution for f_Y and a $N\big(0, 0.8^2\big)$ distribution for f_X. Moreover, in Scenarios 3 and 4, we modeled f_Y as a convolution of a χ^2_3 distribution and a Gamma$(2.25, 0.75)$ distribution, whereas f_X followed a $N(0, 2)$ distribution in Scenario 3 and a $N(0, 10)$ distribution in Scenario 4, representing an increase in variance. To evaluate the methods under disjoint support, we, furthermore, considered two Scenarios 5 and 6 in which the

**Sce.**	σ_Y^2/σ_X^2	n	$\widehat{f}_Y^{\text{NPFD}}$	$\widehat{f}_Y^{\text{DKM}}$	**Sce.**	σ_Y^2/σ_X^2	n	$\widehat{f}_Y^{\text{NPFD}}$	$\widehat{f}_Y^{\text{DKM}}$
**1**	2	500	0.03	0.05	**4**	1	500	0.04	0.27
			[0.02, 0.04]	[0.03, 0.07]				[0.03, 0.05]	[0.26, 0.27]
**2**	15.625	1000	0.02	0.05	**5**	15.969	1000	0.02	0.06
			[0.02, 0.03]	[0.04, 0.06]				[0.02, 0.03]	[0.04, 0.07]
**3**	5	500	0.02	0.05	**6**	16.797	1000	0.05	0.08
			[0.02, 0.03]	[0.05, 0.06]				[0.04, 0.06]	[0.06, 0.09]

Table S2: The median and (in squared brackets) the first and third quartiles of $10 \times$ ISE of the NPFD density estimator $\widehat{f}_Y^{\text{NPFD}}$ and the DKM density estimator $\widehat{f}_Y^{\text{DKM}}$ from 500 simulations.

same densities as in Scenario 2 were considered, but in which we removed from the target density f_Y all probability mass from $[-1, 1]$ or $[-1.5, 1.5]$, respectively.

Since in additive measurement error models the observations of X and Y are paired, we considered $n = n_x = n_z$ in these simulation scenarios. In each of the six scenarios, we assumed, similar to Wang and Wang [2], that we know the error distribution and its exact variance. Instead of using the estimated Fourier transform of f_X, we utilized in NPFD the exact representation of the Fourier transform of a normal or Laplace distribution, as was also done in DKM. For Scenarios 1, 3, and 4, we used the standard values for the parameters provided in the main article. For Scenarios 2, 5, and 6, the target densities present a unique case with two local maxima (see Figures S6 (c) and S7 (a), (c)). When convolved with itself, these density functions transform into a different shape with only one local maximum, leading to a distorted estimation when a power of $N > 1$ is used in NPFD. Therefore, we limited the maximum value $N_{\max}$ to 1 in these scenarios, and chose $\varepsilon = 0.03$ based on the practical diagnostic described in Section S3.3. Even when considering $N_{\max} = 1$, NPFD differs from other deconvolution methods by its numerical procedure presented in Algorithm 1 in the main article. This procedure helps to stabilize the resulting estimator and can still produce reliable estimates even without utilizing a power $N > 1$.

The results presented in Table S2 demonstrate that despite not specifically being designed for the considered situations, the application of NPFD led to favorable estimates of f_Y in comparison to the DKM procedure. Specifically, in Scenario 1 it performed slightly better than the DKM approach in terms of the median, first quartile, and third quartile of $10 \times$ ISE. This difference in performance was even more pronounced for Scenarios 2-6. The plots in Figure S6 show that accurate estimates of the target densities with smooth curves and very small deviations from the true densities were achieved in Scenarios 1 (with $N = 3$) and 2 (with $N = 1$), whereas DKM produced more wiggly curves (see Figure S6 (a), (c)). The results for Scenario 3, which are shown in Figure S6 (e), (f), also indicate a more accurate estimation by NPFD (using $N = 4$), though the improvement over DKM is less pronounced than in Scenario 4. Specifically, in Scenario 4, both methods yield a smooth curve for estimating the target density (see Figure S6 (g)). However, the density estimated by NPFD (using $N = 10$) reproduced the true density f_Y much more closely than the estimated density $\widehat{f}_Y^{\text{DKM}}$. Figure S6 (h) illustrates that this large discrepancy in errors exists in the applications of the two deconvolution methods to all simulated data sets from this scenario.

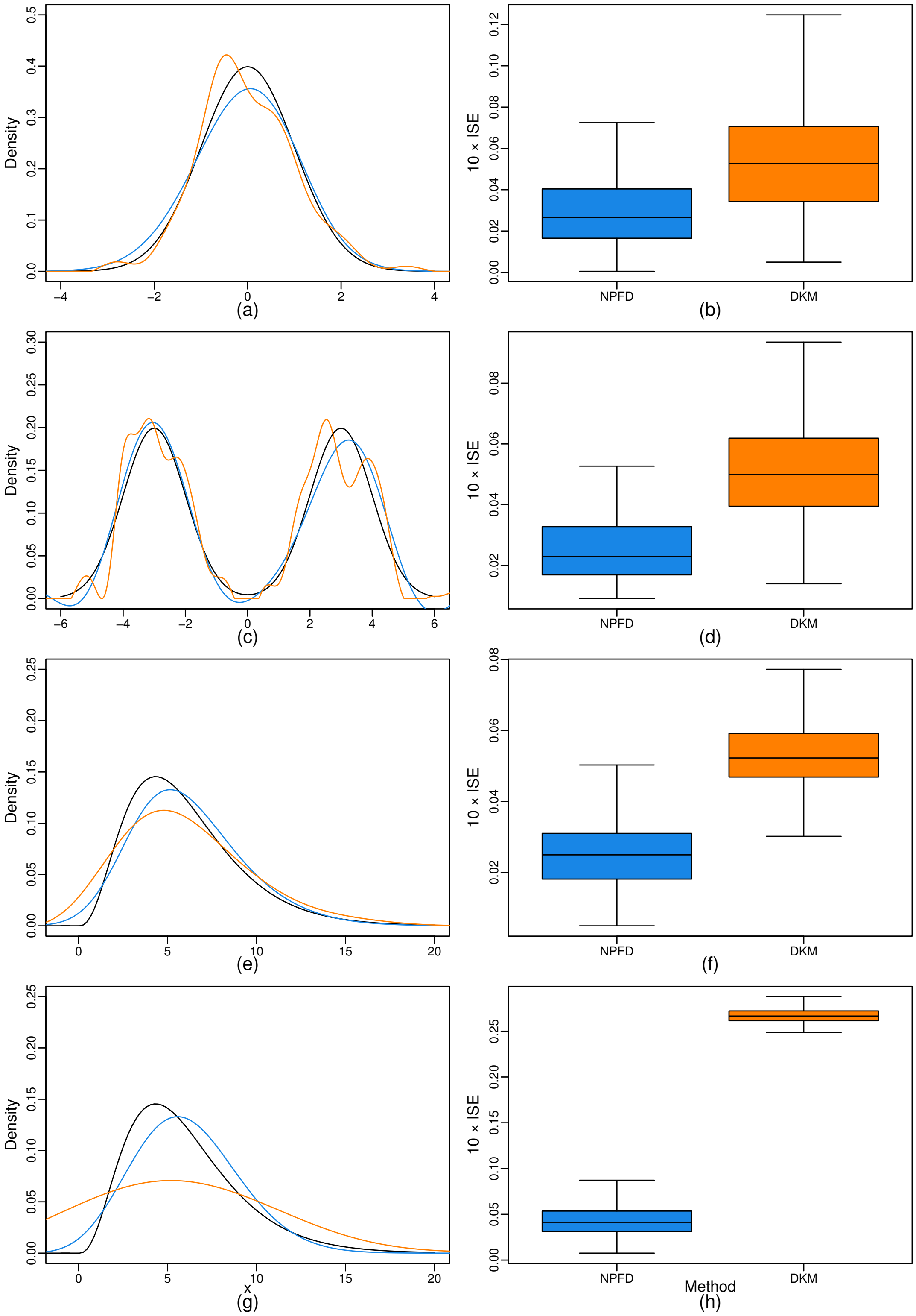

Figure S6: (a), (c), (e), (g): Comparison of a representative density estimate $\widehat{f}_Y^{\mathrm{NPFD}}$ (blue) to a representative density estimate $\widehat{f}_Y^{\mathrm{DKM}}$ (orange) with the true density f_Y (black) for Scenario 1 (a), Scenario 2 (c), Scenario 3 (e), and Scenario 4 (g). (b), (d), (f), (h): Box plots of the values of $10 \times \mathrm{ISE}$ of the density estimators (without outliers) in the corresponding 500 simulated data sets from (a), (c), (e), and (g), respectively.

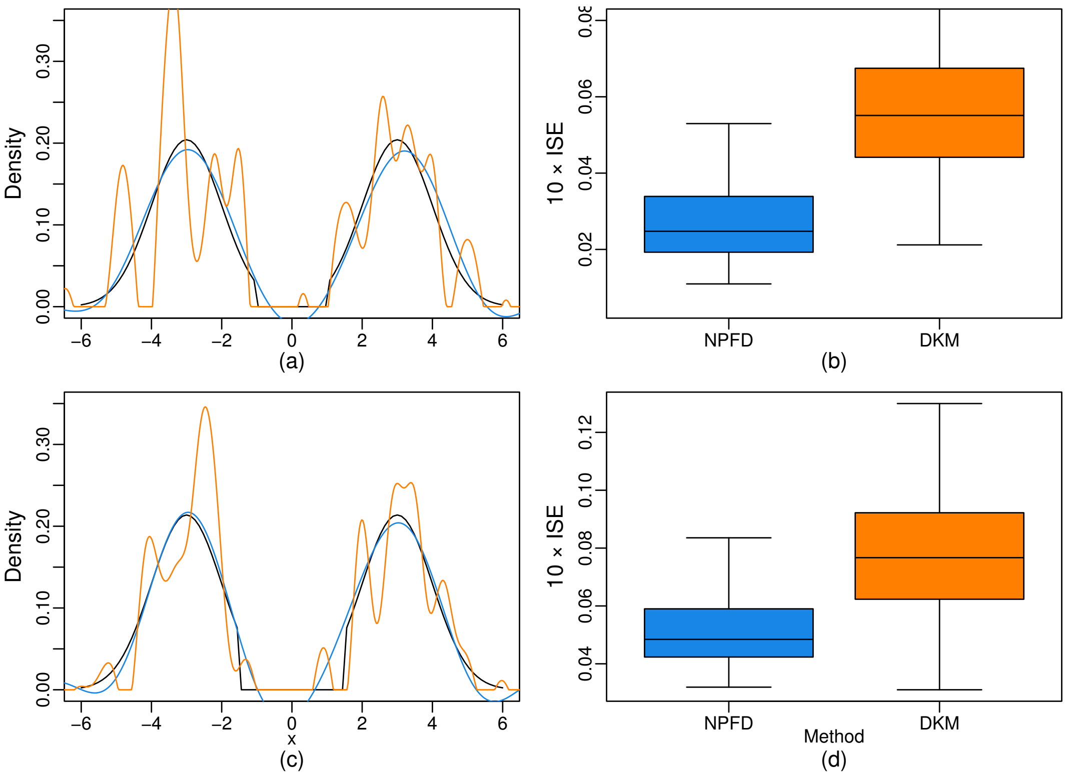

Figure S7: (a), (c): Comparison of a representative density estimate $\widehat{f}_Y^{\mathrm{NPFD}}$ (blue) to a representative density estimate $\widehat{f}_Y^{\mathrm{DKM}}$ (orange) with the true density f_Y (black) for Scenario 5 (a) and Scenario 6 (c). (b), (d): Box plots of the values of 10 × ISE of the density estimators (without outliers) in the corresponding 500 simulated data sets from (a) and (c), respectively.

**Scenario**	N	**Scenario**	N
**1**	3	**4**	10
**2**	1	**5**	1
**3**	4	**6**	1

Table S3: Power values N used in the NPFD estimation for the representative estimate of each scenario in the comparison with DKM.

In Scenarios 5 and 6, in which target densities with distinct supports were considered, NPFD continued to yield accurate and visually smooth estimates (see Figure S7). In particular, NPFD did not perform noticeably worse than in the original example with the bimodal f_Y considered in Scenario 2. In contrast, DKM produced substantially more oscillatory estimates in the scenarios in which probability mass was removed from the support.

The power values N used in the NPFD estimation for the representative estimate of each scenario are given in Table S3.

S4.4 Deconvolution with second experiment data of the convolving distribution

For further investigating the performance of NPFD in the situation in which data from one of the convolving distributions is provided, we considered the two simulation scenarios involving convolved Laplace distributions that were also considered by Neumann [3] to evaluate his density estimator $\widehat{f}_Y^{\mathrm{MCD}}$ described in Section S2.4. Rooted in the idea of convolved Laplace distributions, we considered three additional scenarios. Since the density of a symmetric Laplace distribution is a smooth function in the sense of Section S1.1, deconvolution can be particularly difficult when no assumptions are made about the convolving density when considering data from both the convolving and the mixed distribution.

Let L be a random variable following a standard Laplace distribution with location parameter 0 and scale parameter 1, and let f_L^{*k} denote the k-fold convolution of its density f_L. This convolution can be conveniently characterized via the Fourier transform of f_L, which is given by $\phi_L(t) = 1/(1+t^2)$. Since the Fourier transform of a convolution corresponds to the product of the individual Fourier transforms, the Fourier transform of the k-fold convolution is given by $\phi_{L^{*k}}(t) = 1/(1+t^2)^k$.

In each of the simulation scenarios 1 to 5, the convolution of f_X and f_Y was constructed to yield the same mixed distribution $f_Z = f_L^{*6}$. The order k of the k-fold convolution for the target density f_Y was decreased across scenarios from $k = 5$ to $k = 1$, while the order of the convolving density f_X was increased accordingly from $k = 1$ to $k = 5$, so that, e.g., in Scenario 5, $f_Y = f_L^{*1}$ and $f_X = f_L^{*5}$. Scenarios 2 and 4 correspond to those considered by Neumann [3], while the remaining scenarios were included to systematically extend the analysis.

In the scenarios in which the target density f_Y has a larger fold of convolution, f_Y is more uniform than f_X and Y has a higher variance compared to the convolving variable X. Conversely, in the scenarios in which f_X has a larger convolution power, f_X is smoother than f_Y and X has a larger variance than Y, resulting in more challenging scenarios.

In contrast to the comparison with the method of Diggle and Hall [1], in which equal sample sizes n_x and n_z were considered, we here followed the settings of Neumann [3] and studied scenarios with unequal sample sizes. For each setting, we first considered sample sizes $n_x = 500$ and $n_z = 1000$. As demonstrated by Neumann [3], it is also possible to obtain reliable estimates of the target density f_Y, even if the sample sizes are very small. We, therefore, additionally included the smaller sample size setting $n_x = 10$ and $n_z = 200$.

In NPFD, we estimated the density functions based on the simulated data for the scenarios with the larger sample sizes, while for the smaller sample size, we utilized the empirical Fourier transform method. In the scenarios with the larger sample size, we used the default value $\varepsilon = 0.001$ for the threshold parameter. In order to find the correct bandwidth for situations involving small sample sizes, we employed, as proposed by Neumann [3], $\varepsilon = n_x^{-1/2}$ instead of using $\varepsilon = 0.001$, since this latter value may result in greater inaccuracies for very small sample sizes. All other parameters were kept at their standard values.

In contrast to Diggle and Hall [1], Neumann [3] utilized the exact representation of the Fourier transform of the mixed density to compute the smoothing kernel. This requires prior knowledge of the exact shape of the Fourier transform, which is typically not available. In the application of NPFD, we, hence, completely relied on the technique used in NPFD to approximate the target density without requiring knowledge of the

**Sce.**	σ_Y^2/σ_X^2	n_x	n_z	$\widehat{f}_Y^{\text{NPFD}}$	$\widehat{f}_Y^{\text{MCD}}$
**1**	5	500	1000	0.00	0.00
				[0.00, 0.00]	[0.00, 0.01]
		10	200	0.04	0.05
				[0.02, 0.05]	[0.03, 0.08]
**2**	2	500	1000	0.01	0.01
				[0.00, 0.01]	[0.01, 0.01]
		10	200	0.07	0.08
				[0.04, 0.10]	[0.05, 0.13]
**3**	1	500	1000	0.01	0.03
				[0.01, 0.02]	[0.02, 0.04]
		10	200	0.14	0.16
				[0.10, 0.20]	[0.11, 0.23]
**4**	0.5	500	1000	0.06	0.12
				[0.04, 0.10]	[0.11, 0.15]
		10	200	0.32	0.36
				[0.23, 0.44]	[0.27, 0.51]
**5**	0.2	500	1000	0.42	0.67
				[0.32, 0.56]	[0.62, 0.76]
		10	200	1.05	1.14
				[0.87, 1.22]	[0.97, 1.34]

Table S4: The median and (in squared brackets) the first and third quartiles of $10 \times$ ISE of the NPFD density estimator $\widehat{f}_Y^{\text{NPFD}}$ and the MCD density estimator $\widehat{f}_Y^{\text{MCD}}$ from 500 simulations.

exact Fourier transform of the mixed density.

Table S4 contains summarizing statistics of the values of $10 \times$ ISE for the applications of NPFD and the MCD approach of Neumann [3]. This table demonstrates that NPFD can attain superior outcomes compared to MCD without the additional information used by MCD. NPFD shows smaller errors in each scenario. As the sample size increases, the differences in the amount of error between the two techniques grow larger. This increase of the amount of error is especially noticeable when the ratio σ_Y^2/σ_X^2 or the smoothness of the convolving function increases.

In Figure S8, the results of the density estimations are exemplified considering Scenarios 1 and 2 with large and small sample sizes. The outcomes of Scenario 1 for the large sample sizes, shown in Figure S8 (a), (b), demonstrate that both NPFD and MCD produce good estimates. While the MCD estimator already closely approximated the true density, the NPFD estimator even more closely matched it (see Figure S8 (a)) with a power of $N = 2$. Moreover, Figure S8 (c), (d) shows that NPFD can produce a promising

estimate using the approach based on empirical Fourier transforms even when the sample size is very small. Notably, in this scenario, a power of $N = 1$ was used in NPFD so that no power transformation was necessary in this case to obtain a good density estimation. Using NPFD led to a smooth curve estimation and smaller errors compared to the MCD method in Scenario 1 with smaller sample sizes. In Scenario 2, for which the results are presented in Figure S8 (e), (f) for larger sample sizes and in Figure S8 (g), (h) for smaller sample sizes, a decreased variance of the target variable Y, and thus, an increased variance of the convolving variable X was considered. Despite not utilizing information on the Fourier transform of the mixed density, NPFD with a determined power of $N = 2$ for the large and $N = 1$ for the small sample size setting was able to outperform the MCD estimator and to generate a smooth estimation with smaller errors.

The same applies to the other scenarios, for which the corresponding density estimations and box plots are shown in Figures S9 and S10. The power values N used in the NPFD estimation for each scenario are summarized in Table S5.

**Scenario**	**Sample Sizes**	N
**1**	Large	2
	Small	1
**2**	Large	2
	Small	1
**3**	Large	3
	Small	1
**4**	Large	2
	Small	1
**5**	Large	8
	Small	1

Table S5: Power values N used in the NPFD estimation for the representative estimate of each scenario in the comparison with MCD.

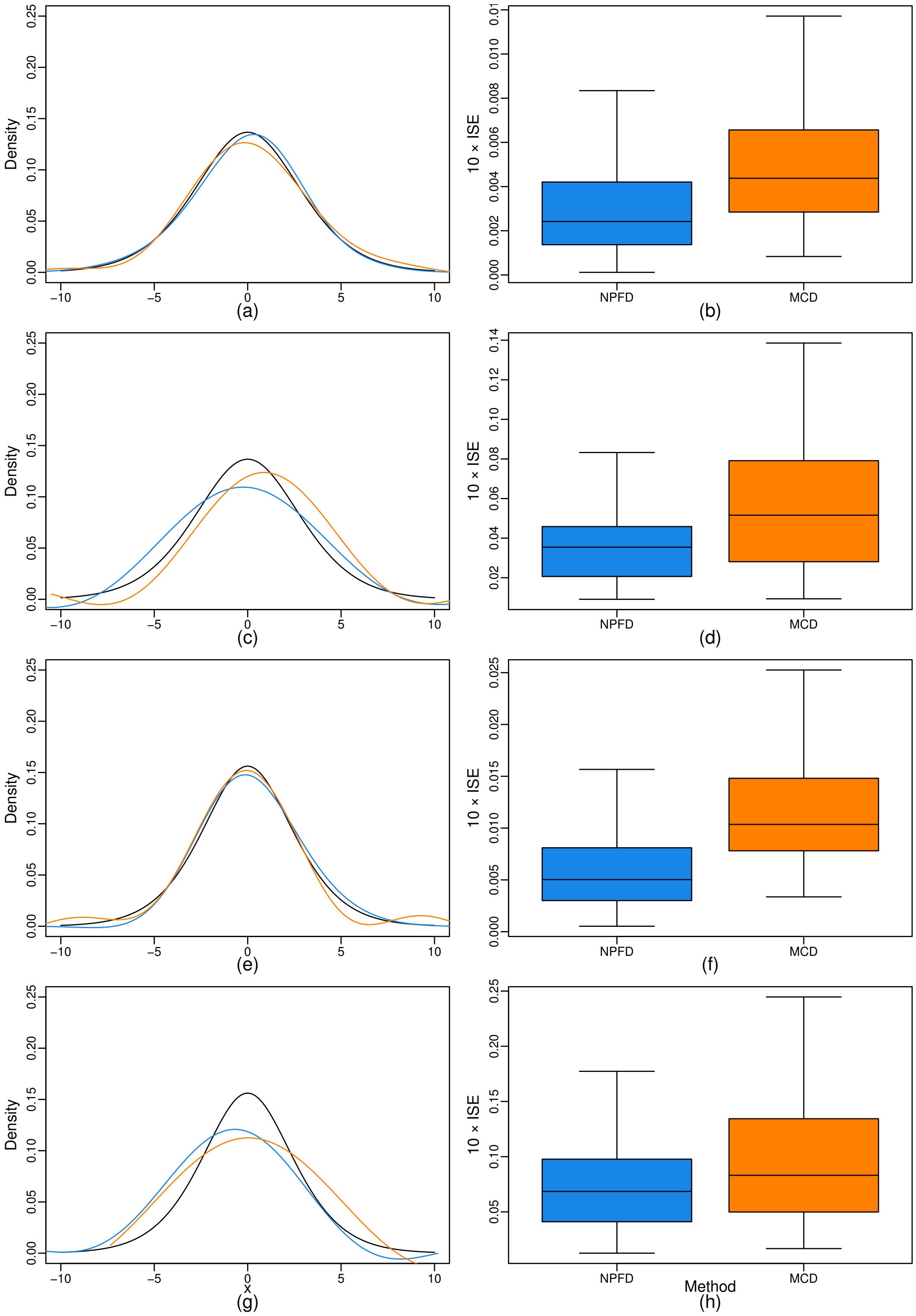

Figure S8: (a), (c), (e), (g): Comparison of a representative density estimate $\widehat{f}_Y^{\mathrm{NPFD}}$ (blue) to a representative density estimate $\widehat{f}_Y^{\mathrm{MCD}}$ (orange) with the true density f_Y (black) for Scenario 1 with large (a) and small (c) sample sizes, as well as for Scenario 2 with large (e) and small (g) sample sizes. (b), (d), (f), (h): Box plots of the values of $10 \times$ ISE of the density estimators (without outliers) in the corresponding 500 simulated data sets from (a), (c), (e), and (g), respectively.

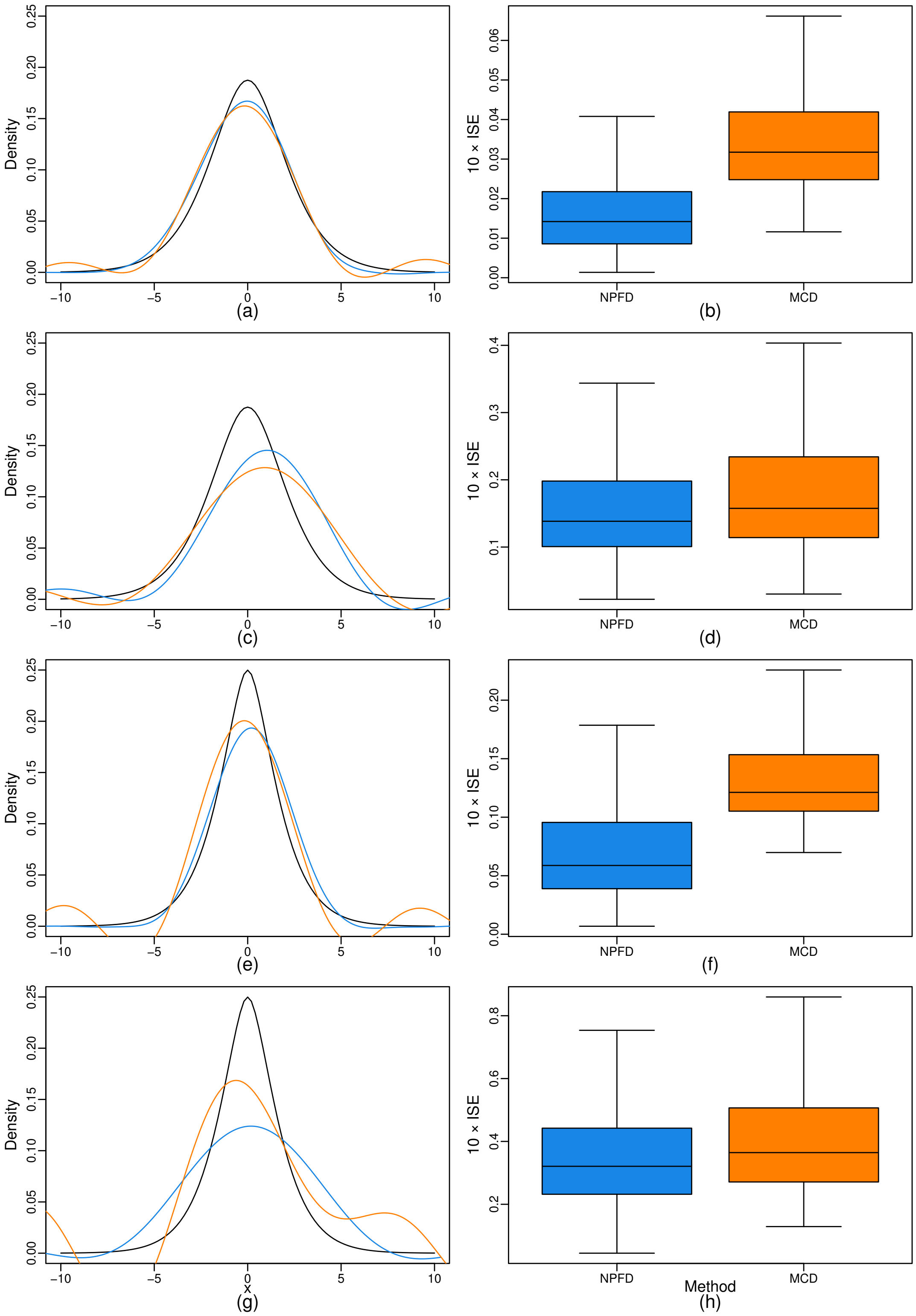

Figure S9: (a), (c), (e), (g): Comparison of a representative density estimate $\widehat{f}_Y^{\mathrm{NPFD}}$ (blue) to a representative density estimate $\widehat{f}_Y^{\mathrm{MCD}}$ (orange) with the true density f_Y (black) for Scenario 3 with large (a) and small (c) sample sizes, as well as for Scenario 4 with large (e) and small (g) sample sizes. (b), (d), (f), (h): Box plots of the values of $10 \times$ ISE of the density estimators (without outliers) in the corresponding 500 simulated data sets from (a), (c), (e), and (g), respectively.

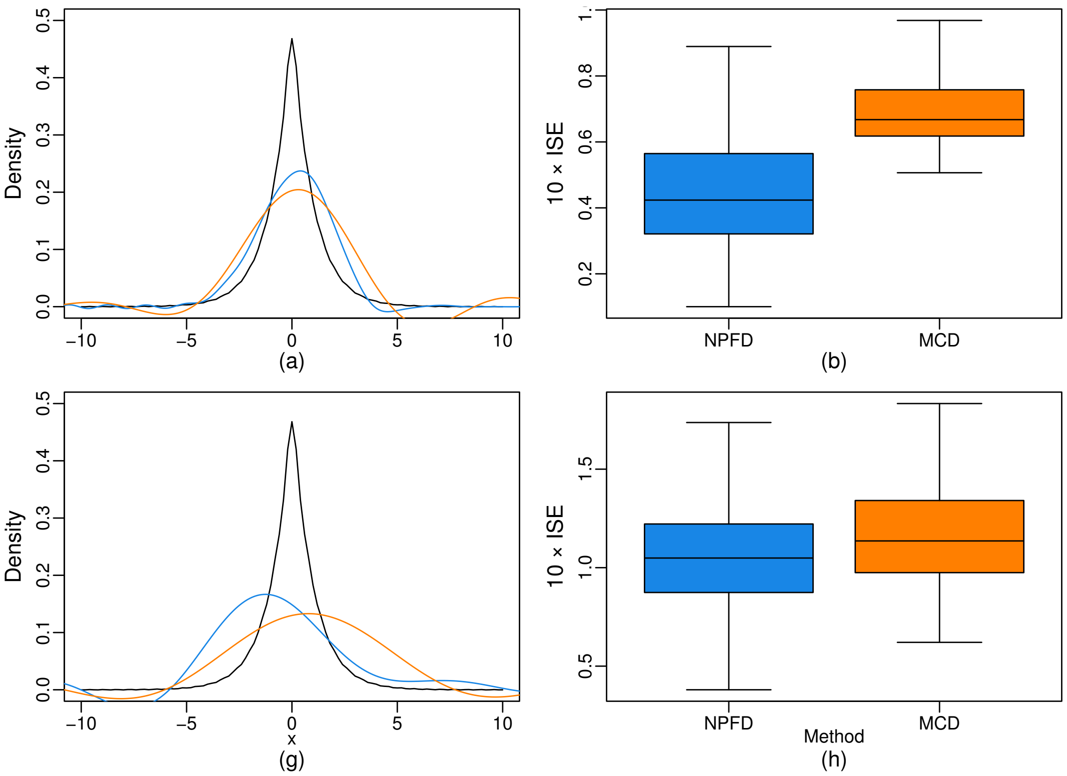

Figure S10: (a), (c): Comparison of a representative density estimate $\widehat{f}_Y^{\mathrm{NPFD}}$ (blue) to a representative density estimate $\widehat{f}_Y^{\mathrm{MCD}}$ (orange) with the true density f_Y (black) for Scenario 5 with large (a) and small (c) sample sizes. (b), (d): Box plots of the values of $10 \times \mathrm{ISE}$ of the density estimators (without outliers) in the corresponding 500 simulated data sets from (a) and (c), respectively.

S5 Application of FDD to the Proteomic Data

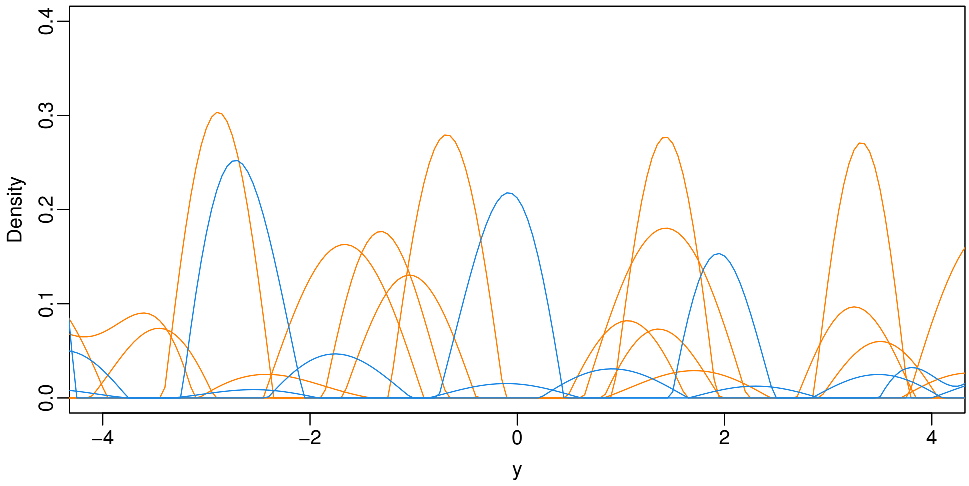

Figure S11: Deconvolved densities of the extrinsic signal of the proteins from the proteome of the skin fibroblasts for the five women of the younger age group (marked by orange lines) and four women of the older age group (blue lines) from the GerontoSys study.

References

[1] Diggle PJ, Hall P. A Fourier approach to nonparametric deconvolution of a density estimate. J R Stat Soc Ser B Methodol. 1993;55(2):523–531.

[2] Wang XF, Wang B. Deconvolution estimation in measurement error models: the R package decon. J Stat Softw. 2011;39(10):1–24.

[3] Neumann MH. On the effect of estimating the error density in nonparametric deconvolution. J Nonparametr Stat. 1997;7(4):307–330.

[4] Waldera-Lupa DM, Kalfalah F, Florea AM, Sass S, Kruse F, Rieder V, et al. Proteome-wide analysis reveals an age-associated cellular phenotype of in situ aged human fibroblasts. Aging (Albany NY). 2014;6(10):856–878.

[5] Rudin W. Real and complex analysis. 3rd ed. New York: McGraw-Hill; 1987.

[6] Delaigle A, Hall P. Methodology for nonparametric deconvolution when the error distribution is unknown. J R Stat Soc Ser B Stat Methodol. 2016;78(1):231–252.

[7] Fan J. On the optimal rates of convergence for nonparametric deconvolution problems. Ann Stat. 1991;19(3):1257–1272.

[8] Nolan JP. Numerical calculation of stable densities and distribution functions. Commun Stat Stoch Models. 1997;13(4):759–774.

[9] Shaked M, Shanthikumar JG. Stochastic Orders. 1st ed. New York: Springer Science+Business Media; 2007.

[10] Stefanski L, Carroll RJ. Deconvoluting kernel density estimators. Stat. 1990;21:169–184.

[11] Delaigle A, Gijbels I. Bootstrap bandwidth selection in kernel density estimation from a contaminated sample. Ann Inst Stat Math. 2004;56(1):19–47.

[12] Delaigle A, Hall P, Meister A. On deconvolution with repeated measurements. Ann Stat. 2008;36(2):665–685.

[13] Bartlett MS. Periodogram analysis and continuous spectra. Biometrika. 1950;37(1–2):1–16.

[14] Efron B, Tibshirani R. Using specially designed exponential families for density estimation. Ann Stat. 1996;24:2431–2461.

[15] Schwender H, Ickstadt K. Empirical Bayes analysis of single nucleotide polymorphisms. BMC Bioinformatics. 2008;9(1):144.

[16] Scott DW. On optimal and data-based histograms. Biometrika. 1979;66(3):605–610.

[17] Jones MC. The roles of ISE and MISE in density estimation. Stat Probab Lett. 1991;12(1):51–56.

[18] Wand MP. Data-based choice of histogram bin width. Am Stat. 1997;51(1):59–64.

[19] Quarteroni A, Sacco R, Saleri F. Numerical mathematics. 2nd ed. Berlin, Heidelberg: Springer; 2007. (Texts in Applied Mathematics; vol. 37).

[20] Delaigle A, Hyndman T, Wang T. deconvolve: Deconvolution tools for measurement error problems [R package]. Version 0.1.0. 2024. Available from: https://github.com/TimothyHyndman/deconvolve
